# A catalyst-aware explainable machine learning framework for biodiesel yield prediction over metal-doped biochar and activated carbon catalysts

**DOI:** 10.1039/d6ra05362a

**Published:** 2026-08-05

**Authors:** Menna Ebrahim, Mostafa Mahmoud, Fatma H. Ashour, Osama A. Ibrahim, Nourhan H. Khashaba

**Affiliations:** a Chemical Engineering Department, Faculty of Engineering, Cairo University Giza 12613 Egypt menna.202211310@eng-st.cu.edu.eg; b Public Work Department, Faculty of Engineering, Cairo University Giza 12613 Egypt

## Abstract

Biodiesel production over metal-doped biochar and activated carbon (AC) catalysts involves complex nonlinear interactions among feedstock characteristics, catalyst descriptors, and operating conditions, making accurate yield prediction a challenging task. While machine learning (ML) has shown potential in process modeling, existing studies lack catalyst-aware frameworks that integrate material and process descriptors within a unified representation for biodiesel yield prediction. Furthermore, current approaches are often limited by small datasets and insufficient model interpretability, restricting their ability to support reliable catalyst screening and process optimization. To address these challenges, this study develops an explainable ML framework for biodiesel yield prediction using a literature-derived dataset of metal-doped biochar and AC catalyst systems. The framework integrates catalyst, feedstock, and operating-condition descriptors, augments sparse experimental data through curve digitization, evaluates six ML models, and applies SHAP and CatBoost-based explainability analysis. The neural network model achieved the highest predictive accuracy on unseen data, with RMSE of 3.27%, MAE of 1.64%, and *R*^2^ of 0.95, whereas linear regression showed the weakest performance, highlighting the nonlinear behavior of the catalytic system. Validation using an independent experimental dataset further confirmed model generalization. Explainability analysis identified alcohol-to-oil ratio, reaction time, catalyst amount, reaction temperature, and feedstock acid value as the key factors governing biodiesel yield. The proposed ML framework provides an accurate and interpretable approach for catalyst screening and data-driven optimization of sustainable biodiesel production processes.

## Abstract 2

An explainable catalyst-aware machine learning framework predicts biodiesel yield over metal-doped biochar and activated carbon catalysts and reveals the key variables controlling performance.

## Graphical abstract

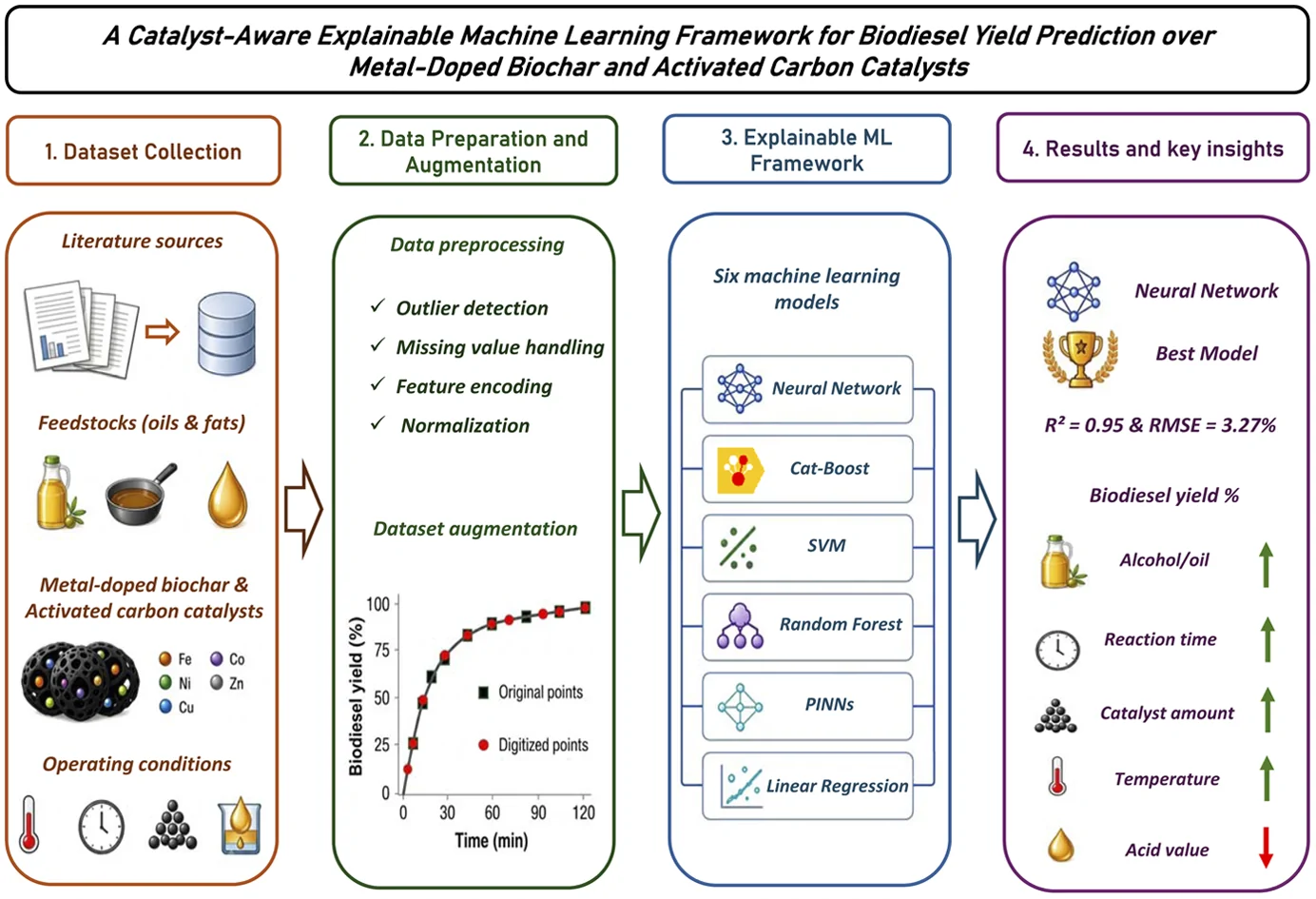

## 1. Introduction

Biodiesel is a renewable liquid biofuel composed primarily of fatty acid methyl esters (FAMEs) and is produced through chemical conversion processes, mainly esterification and transesterification, using diverse lipid-based feedstocks such as vegetable oils, waste cooking oils, animal fats, and other renewable resources.^1^ Its continued development is driven by the need to reduce dependence on petroleum-derived diesel while improving the sustainability profile of transport fuels. At the same time, biodiesel production remains highly sensitive to feedstock quality, catalyst properties, alcohol-to-oil ratio, reaction temperature, reaction time, and catalyst loading. These coupled effects motivate the search for catalyst systems that are active, reusable, inexpensive, and suitable for low-quality feedstocks.^2^ In particular, high free fatty acid and moisture contents in low-quality feedstocks can reduce process efficiency and often require pretreatment before transesterification.^3^

Within this context, waste-derived AC and biochar have become attractive catalyst supports because they provide porous carbon frameworks that can anchor acid or base functionalities and can be produced from low-value biomass residues.^4^ Metal doping further enhances these materials by increasing the density of active sites, improving mass transfer, and enabling functionalities such as bifunctionality or magnetic recovery.^5^ Nevertheless, metal-doped biochar systems also raise practical questions related to metal leaching, pore blockage, feedstock-dependent performance, and the trade-off between catalyst complexity and process stability.^6^ Biochar is especially attractive because it is a porous carbon-rich material obtained from biomass pyrolysis, but pristine biochar often shows limited catalytic activity unless it is modified or doped.^7^ As a result, biodiesel yield in such systems is governed not only by operating conditions, but also by catalyst physicochemical characteristics such as support type, metal loading, and surface properties, which complicates both catalyst screening and process optimization.

Modeling offers a complementary route for accelerating catalyst and process development. Conventional kinetic, reactor-based, and semi-empirical models remain valuable when reaction pathways are well characterized, and data are generated under controlled experimental conditions. However, their broader applicability becomes limited when biodiesel data are compiled from multiple literature sources and when strong nonlinear interactions exist among catalyst descriptors, feedstock properties, and operating variables.^8^ This limitation becomes more important in biodiesel systems based on waste-derived carbon catalysts, where yield depends not only on operating conditions but also on catalyst physicochemical properties such as surface area, pore structure, and metal loading.^9^ As a result, data-driven approaches have become increasingly attractive for biodiesel modeling because they can capture complex nonlinear relationships without requiring a complete mechanistic description of the system.^10^

ML approaches have therefore been increasingly adopted for biodiesel applications, including property prediction, process optimization, and yield estimation.^11^ Prior studies demonstrate that neural-network, support-vector, and hybrid models can achieve high accuracy in biodiesel-related tasks; however, they are still limited by small datasets, narrow catalyst scope, and low interpretability.^12^ More critically, to the best of our knowledge, no previous study has developed a catalyst-aware ML model for biodiesel-yield prediction over metal-doped biochar and activated carbon catalysts. In addition, the literature does not yet provide a clear and unified way to represent the key input features, as catalyst descriptors, feedstock descriptors, and operating conditions are often incomplete, fragmented, or not explicitly encoded within a single predictive framework.^13^ As a result, an explainable model that combines heterogeneous literature data with an appropriate feature representation for biodiesel-yield prediction is still lacking.^14^

To address these limitations, this study develops a catalyst-aware explainable machine learning framework for biodiesel yield prediction using a literature-derived dataset focused on metal-doped biochar and activated carbon catalysts under varying reaction conditions. The main contributions of this work are as follows:

- Developing an interpretable catalyst-aware ML framework that integrates catalyst characteristics, feedstock properties, and operating conditions for biodiesel yield prediction.

- Constructing and enriching a literature-derived dataset through unified feature representation and curve-digitization-based reconstruction of experimental trends to improve data coverage and model learning.

- Systematically benchmarking six ML models under a consistent evaluation protocol to identify the most reliable prediction approach.

- Assessing model generalization using an independent experimental holdout dataset beyond the training data.

- Applying explainability analysis to reveal the key factors controlling biodiesel yield and provide insights for catalyst screening and process optimization.

The scientific contribution of this study lies not merely in applying machine learning algorithms, but in developing an integrated catalyst-aware and explainable framework that combines heterogeneous experimental data, meaningful feature representation, rigorous validation, and interpretable analysis to reveal the relationships between catalyst characteristics, reaction conditions, and biodiesel yield.

The remainder of this paper is organized as follows. Section 2 reviews relevant catalyst and ML literature. Section 3 describes data collection and methodology, including preprocessing, augmentation, model selection, training protocol, evaluation metrics, and explainability tools. Section 4 presents the results and discussion, and Section 5 summarizes the main conclusions.

## 2. Literature review

### 2.1 Metal-doped biochar catalysts for biodiesel production

Carbonaceous catalysts derived from agricultural residues, food-processing waste, shell waste, and other biomass streams have been widely investigated for biodiesel applications because they are inexpensive, porous, and compatible with circular-economy strategies.^4^ When these materials are doped or supported with metals such as Ca, K, Fe, Ni, Cu, or Mn, the resulting catalyst can offer stronger catalytic sites, easier recovery, and improved performance across esterification and transesterification reactions. The catalytic role of metal-doped biochar is therefore not limited to passive support effects; instead, the carbon matrix and the metal phase act together to determine active-site accessibility, tolerance to feedstocks with high free fatty acid (FFA) content, and catalyst stability. At the same time, literature on these systems repeatedly identifies leaching, insufficient long-term durability testing, and inconsistent reporting of physicochemical descriptors as important barriers to broader industrial transfer.^6^ These recurring limitations are especially important because they affect not only catalyst lifetime, but also the reliability of any predictive framework intended to relate catalyst formulation to biodiesel yield.

Metal-doped biochar catalysts are commonly prepared either by *in situ* addition of the metal precursor before pyrolysis or by post-treatment impregnation of the prepared biochar, where the first route is simpler and more scalable and the second allows better control over metal dispersion and particle characteristics.^9^ Reported systems doped with K, Ca, Ni, or mixed metals have achieved high fatty acid methyl ester yields across different oils and operating conditions, in many cases exceeding 96%.^15^ In addition, metal incorporation can improve catalyst performance by increasing the density and strength of active sites and by modifying pore structure and accessible surface area.^16^ Some metal-doped systems also provide practical benefits such as magnetic recovery and bifunctionality, although metal leaching, pore blockage, catalyst deactivation, and limited durability assessment remain key challenges.^9^ Accordingly, the catalyst literature suggests that the key issue is not only whether metal doping improves biodiesel yield, but also how catalyst physicochemical properties, preparation route, and stability jointly govern performance across different feedstocks and operating conditions.

### 2.2 Machine learning applications for biodiesel production

The available literature shows that ML models have already been used successfully in two broad biodiesel-related domains: (i) prediction of biodiesel properties such as viscosity, density, oxidation stability, and cold-flow behavior, and (ii) prediction or optimization of biodiesel yield. Table 1 summarizes the representative ML studies reported in biodiesel research.^17^ Neural-network models are common in the literature, while adaptive neuro-fuzzy inference systems (ANFIS), least-squares support-vector methods, and hybrid optimization-coupled algorithms are also reported.^11,12,18–25^ However, most previous studies remain focused on conventional catalyst systems, single feedstocks, or narrowly defined experimental campaigns. In most cases, the predictive inputs are dominated by feedstock and operating variables, whereas catalyst-specific physicochemical descriptors are included far less consistently. Therefore, the literature provides a strong methodological foundation but not yet a generally explainable framework specific to metal-doped biochar catalysts.

**Table 1:** Representative machine learning studies reported in biodiesel research

Application	Main inputs	Model	Representative outcome	Ref.
NIR-based biodiesel property prediction	Spectroscopic variables	MLPNN	RMSE ∼0.068–0.51 for density, viscosity, water, and methanol	11
Oxidative stability and fuel property modeling	FAME descriptors	MLPNN	*R* ∼0.99 for viscosity, iodine value, and induction period	12
Biodiesel property estimation	Temperature, FA%, molar mass	ANFIS	Reliable nonlinear property prediction	18
Biodiesel property estimation	FAME composition	PSO-LSSVM	Slightly better than ANFIS in some property tasks	19
Waste cooking oil process optimization	Catalyst dosage, alcohol/oil ratio, time, stirring, and impurities	SVM-GA	RBF-SVM gave the best predictive accuracy and supported optimization	20
Microwave biodiesel kinetics	Process variables + mechanistic constraints	PINN	Physics-informed, kinetically consistent prediction	21
Soybean oil biodiesel yield	*T*, time, catalyst%, alcohol/oil ratio	MLPNN	*R* ^2^ ∼ 0.996	22
Castor oil biodiesel yield	Process conditions	ANN	Yield prediction with kinetic parameter estimation	23
Soybean biodiesel yield comparison	Catalyst%, alcohol/oil ratio, time	ANN *vs.* LR	ANN outperformed linear regression	24
Jatropha-algae blend optimization	Process variables	ANN *vs.* RSM	ANN outperformed response surface methodology	25

Collectively, these studies show that ML is effective for biodiesel prediction and optimization, but most are based on single-feedstock, single-catalyst, or otherwise narrowly defined datasets and do not explicitly incorporate catalyst physicochemical descriptors relevant to metal-doped biochar systems. More broadly, ML has also been applied to biodiesel process optimization and fuel-property prediction using neural networks, support vector machine (SVM), random forest or random tree methods, and hybrid optimization approaches such as genetic algorithms (GA), particle swarm optimization (PSO), and Bayesian optimization.^26,27^ However, most of these studies still rely on relatively small datasets, emphasize internal validation, and provide limited interpretability.^13,28^ In addition, catalyst physicochemical descriptors are rarely integrated systematically into predictive frameworks, despite their central role in determining catalyst performance.^9,10^

More importantly, prior biodiesel ML studies rarely combine catalyst descriptors, feedstock descriptors, operating conditions, multi-source literature data, and explainability analysis within a single unified framework. This limitation is especially important for metal-doped biochar and AC catalysts, where biodiesel yield depends not only on reaction conditions but also on catalyst-specific properties such as metal loading, surface area, and support characteristics. As a result, the current literature provides a useful methodological foundation, but it does not yet offer a catalyst-aware, explainable ML framework tailored to heterogeneous literature-derived biodiesel datasets.

To this end, this study develops an explainable machine learning framework for biodiesel production over metal-doped biochar and activated carbon catalysts, with a catalyst-aware and experimentally grounded modeling strategy that distinguishes it from generic machine learning applications. The framework focuses on biodiesel yield prediction and feature importance analysis, using an augmented literature-derived dataset that integrates catalyst, feedstock, and operating condition descriptors into a unified modeling structure. Multiple regression-based machine learning models are benchmarked, and explainability analysis is applied to identify the key variables governing biodiesel yield.

## 3. Methodology

**Fig. 1:**
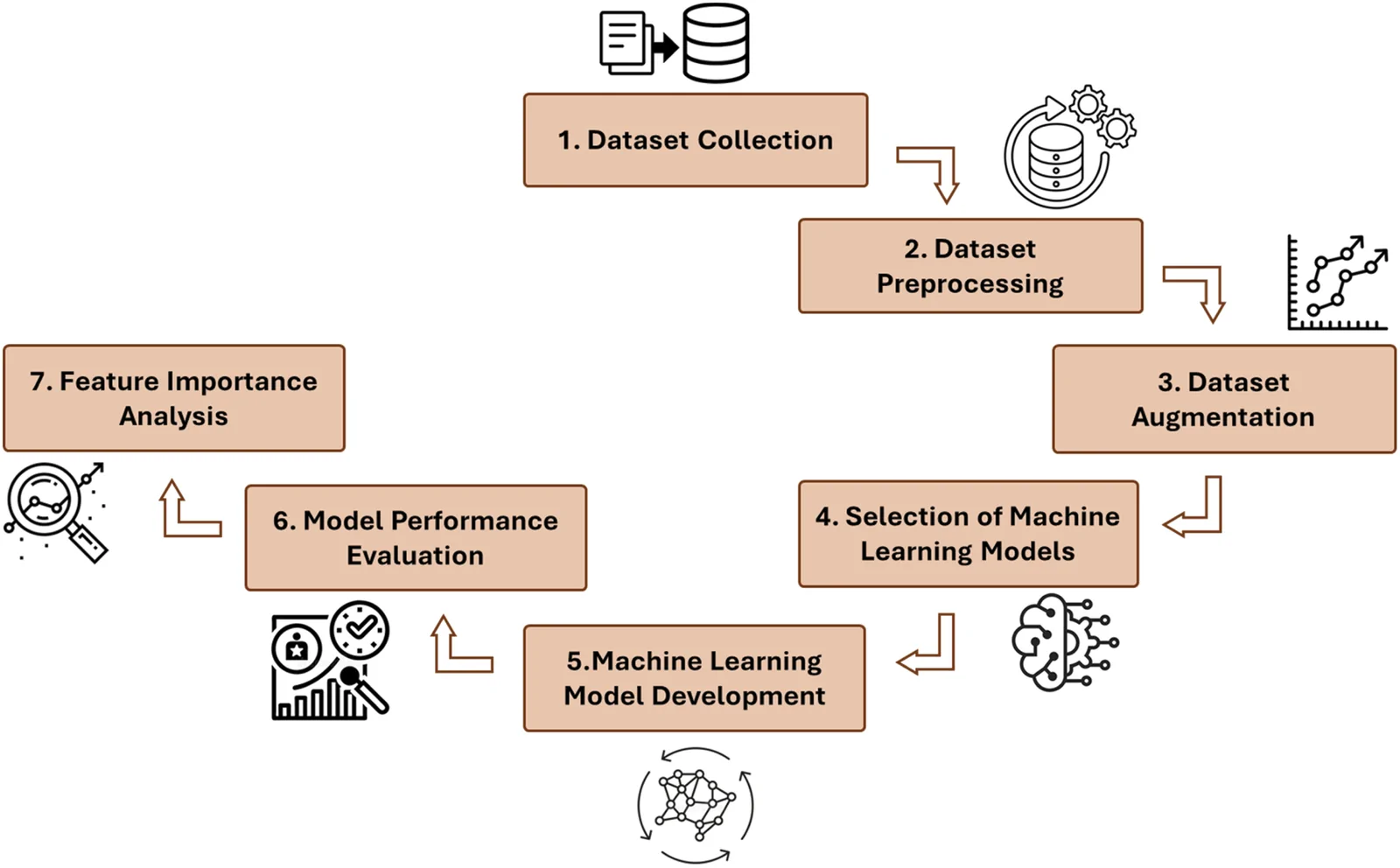
Proposed methodology workflow for explainable yield prediction.

Fig. 1 presents the overall methodology used in this study for biodiesel yield prediction. The workflow consists of six main steps: dataset preprocessing, dataset augmentation, selection of ML models, model training, performance evaluation, and feature importance analysis. First, the dataset was cleaned, normalized, and prepared for modeling. Then, the data were augmented to increase the number of usable observations. After that, several ML models were selected and compared. The models were trained and validated using a structured protocol, and their predictive performance was evaluated using standard statistical metrics. Finally, feature importance analysis was carried out to identify the variables that most strongly influence biodiesel yield.

### 3.1 Dataset collection

The dataset used in this study was collected from the literature rather than generated through a new laboratory campaign. This choice was motivated by the need to cover a broader combination of feedstocks, catalyst chemistries, and operating windows that would be feasible in a single experimental program. Using published studies also enabled the work to leverage existing knowledge while avoiding the cost and time required for additional experiments. In the companion review effort, literature identification followed a structured Scopus-based search, exclusion screening, and snowballing workflow, yielding a broad evidence base on metal-doped AC and biochar catalysts. From that larger body of work, studies containing extractable biodiesel-yield data and sufficient process descriptors were selected for dataset construction.

The dataset used in this study is composed of 27 independent literature-based experimental studies, each representing a distinct catalyst–feedstock system. This dataset spans 13 feedstock categories, 15 metal categories, and 20 biochar/support-source categories, demonstrating that the dataset is materially diverse even though it remains literature-derived. Each data point record was designed to pair one biodiesel-yield value with the feedstock descriptors, catalyst descriptors, and process variables reported for that condition. The initial candidate variables included feedstock oil, acid value, feedstock FFA, water content, metal type, biochar source, metal loading, surface area, pore volume, pore size, alcohol-to-oil molar ratio, reaction temperature, reaction time, catalyst amount, mixing intensity, and biodiesel yield. As outlined in Table 2, the fifteen input variables and the yield output collected for modeling were selected to represent the key process conditions and material properties relevant to biodiesel yield prediction.

**Table 2:** Input and output variables collected for the modeling workflow

Group	Variable	Type	Status
Feedstock descriptors	Feedstock oil, acid value, FFA	Mixed	Retained
Catalyst descriptors	Metal type, biochar source, metal load, surface area	Mixed	Retained
Operating conditions	Alcohol/oil ratio, temperature, time, catalyst amount, mixing	Numerical	Retained
Sparse variables	Water, pore volume, pore size	Numerical	Removed
Target	Biodiesel yield	Numerical	Output

### 3.2 Dataset preprocessing

The modeling data were organized as a structured spreadsheet in which each row represents one biodiesel-yield observation, and each column corresponds to an input descriptor or target value. The original dataset consists of 252 experimentally reported data points. These variables are classified as input variables and one output variable. Preprocessing started with inspection of variable names, unit consistency, category spelling, and cell formatting. This step was necessary because literature-derived data typically contain inconsistent capitalization, white-space artifacts, and mixed reporting conventions across source studies.

Missing-data treatment followed two complementary rules. Variables that were broadly absent across the literature (water content, pore volume, and pore size) were removed from the final feature set. Remaining sparse missing entries were handled within the preprocessing pipeline through imputation so that models could be trained on a complete matrix. For numerical variables, imputation and scaling were applied where required by the model family, whereas categorical variables were encoded before fitting models such as support vector machine, linear regression, and the neural network. In the neural-network workflow, numerical imputation and scaling were used together with one-hot encoding for categorical inputs. CatBoost was treated differently because it can natively handle mixed feature types and includes built-in mechanisms for processing categorical variables.

The acid value of the feedstock (mg KOH per g) and free fatty acid content (feedstock FFA%) were treated as interchangeable descriptors. When one of these variables was missing, but the other was available, the missing value was calculated using standard conversion relationships to avoid unnecessary data loss. The conversion is given by:1
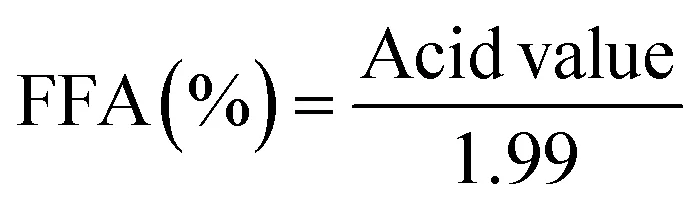


This approach allowed recovery of incomplete records and improved dataset completeness before model training, while maintaining chemical consistency by assuming oleic acid as the representative fatty acid.^29^

To ensure valid model evaluation and avoid data leakage, dataset splitting was performed at the study level rather than at the individual data-point level. Specifically, all digitized points originating from the same experimental study were kept within the same subset. Accordingly, 25 studies (approximately 90% of the dataset) were used for model training and internal testing, while 2 independent studies (approximately 10%) were reserved as an unseen holdout set for evaluation. This strategy ensures that no data from the same experimental study appears in both training and testing sets, preserving the independence of validation.

### 3.3 Dataset augmentation

The initial dataset was compiled directly from experimental studies reported in the literature. For each experimental run, the available information typically consisted of a single biodiesel yield value measured at discrete sampling times, together with the corresponding process descriptors, including feedstock properties, catalyst characteristics, and reaction conditions. Because the number of available samples was limited, the original dataset was sparse and insufficiently dense to describe the nonlinear relationships governing the process. As a result, preliminary ML models showed weak generalization ability, unstable training behavior, and relatively high prediction errors.

To address this limitation, a dataset augmentation strategy based on published experimental curves was adopted. This step was motivated by the need to increase data density so that the models could better capture nonlinear relationships in the process. Sparse experimental points can limit the learning of reaction kinetics, reduce model robustness, and lead to high prediction errors. Therefore, instead of relying only on the originally reported discrete measurements, additional numerical points were generated from the published experimental curves.

**Fig. 2:**
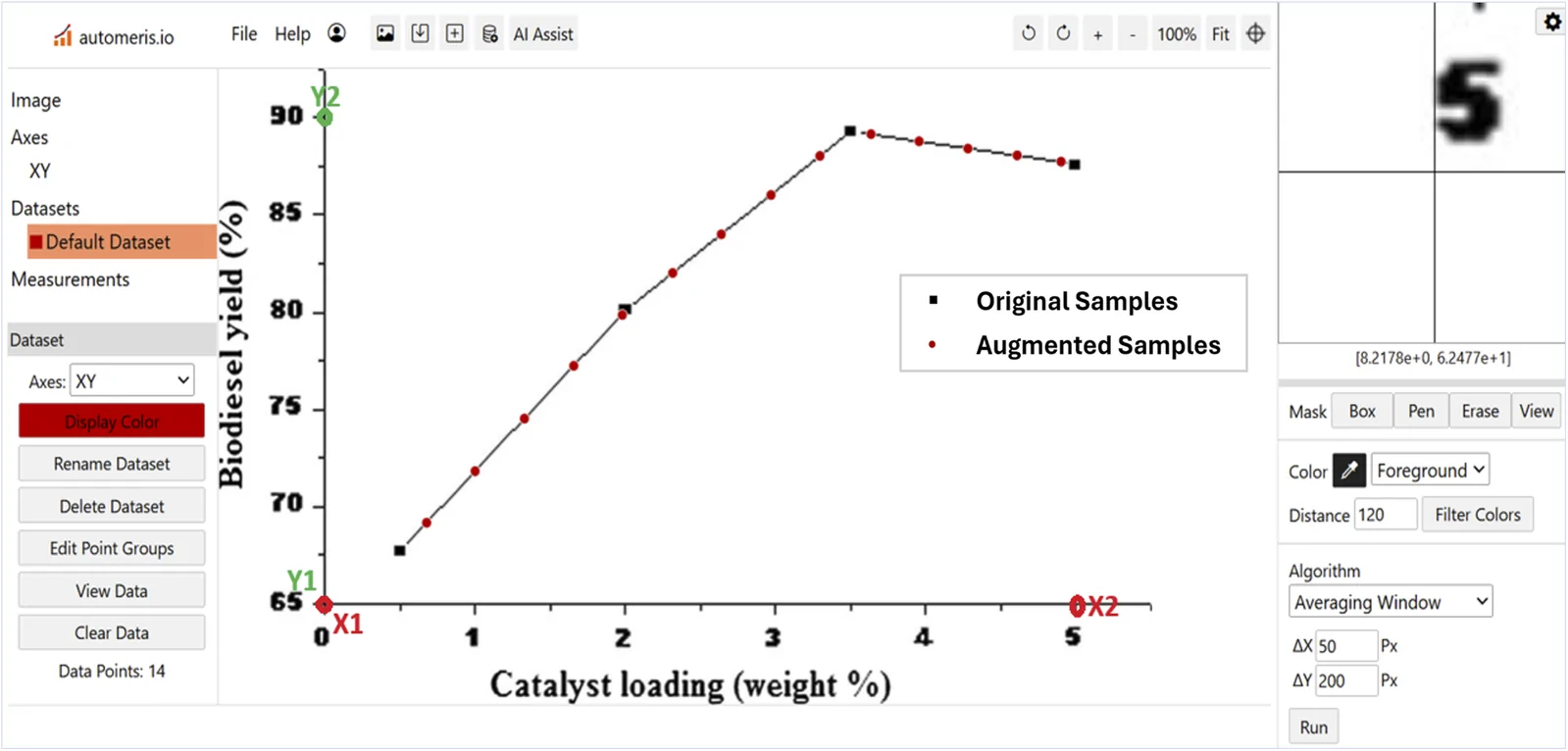
Screenshot of training data augmentation process.

The augmentation workflow followed the following procedure. Relevant graphs (*e.g.*, yield *vs.* time or catalyst loading) were extracted from the literature and digitized using WebPlotDigitizer after axis calibration. Data points were then obtained at finer intervals (*e.g.*, 1 min instead of 5 min), exported as CSV files, and merged with the original dataset while preserving all experimental conditions (feedstock properties, catalyst characteristics, and reaction parameters). Fig. 2 illustrates the curve-digitization augmentation process for a representative case showing the relationship between catalyst loading (wt%) and biodiesel yield (%). The black square markers represent the original input data, whereas the red circular markers represent the augmented data generated from the digitized curve.

**Fig. 3:**
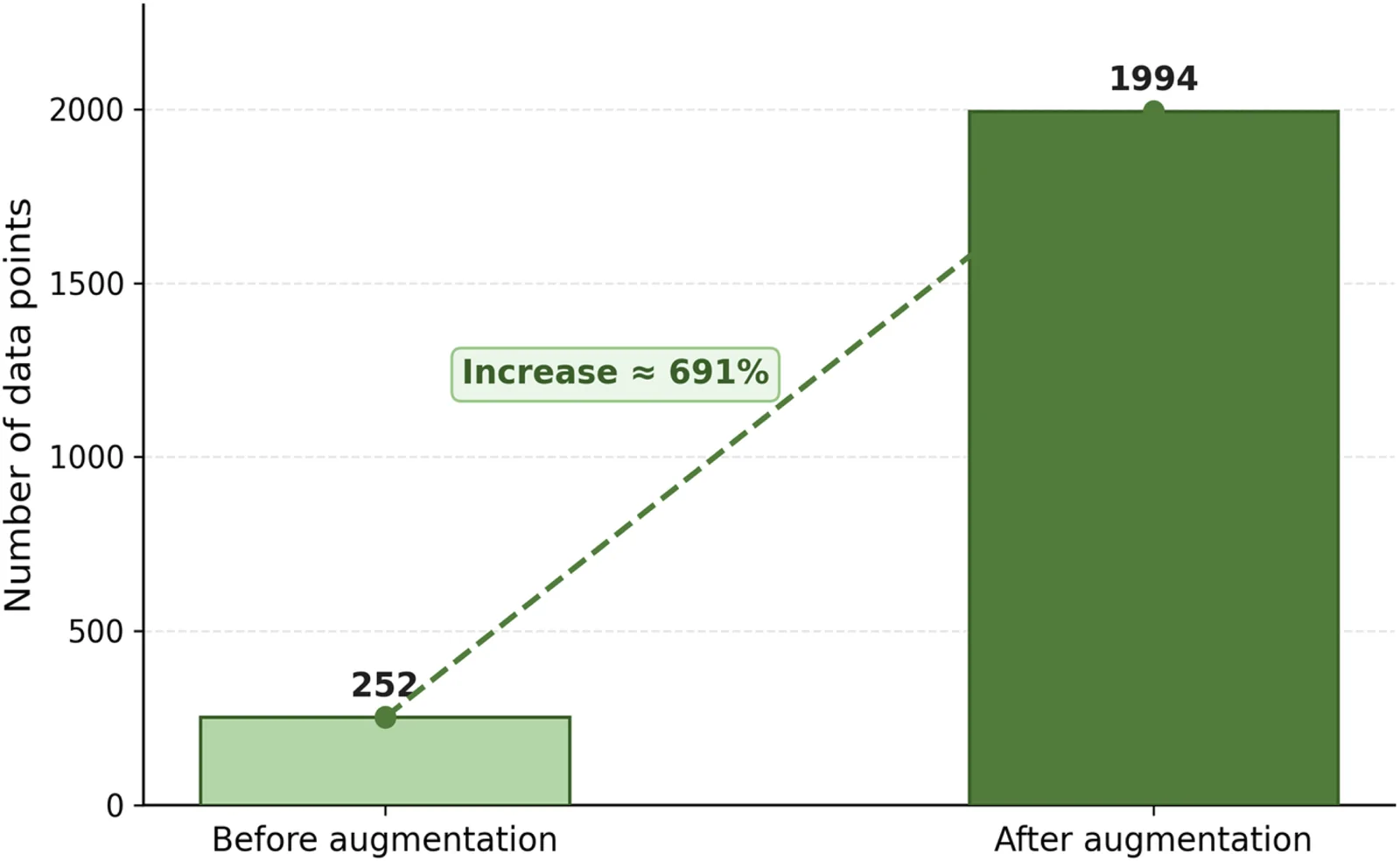
Growth of the dataset after literature-curve digitization.

Curve digitization was employed to extract additional data points from published experimental trends, thereby increasing the usable dataset from 252 original experimental observations to 1994 reconstructed data points, corresponding to an approximately 691% expansion, as shown in Fig. 3. The dataset was partitioned such that the training studies comprised a total of 1794 data points used for model training and internal testing, while the evaluation studies comprised 200 data points reserved as an unseen holdout set for final model evaluation.

The proposed augmentation procedure enhances the temporal and parametric resolution of the reaction behavior by providing a denser representation of yield evolution and process dynamics across the studied conditions. However, it is important to note that these digitized points are not independent experiments; rather, they represent continuous measurements derived from the same underlying literature studies. Accordingly, the expanded dataset improves the representation of reaction kinetics and nonlinear process behavior while remaining fully consistent with the original experimental observations reported in the source literature.

The denser dataset also improved model-training stability and reduced overfitting. Consequently, the predictive models showed improved accuracy and higher reliability in biodiesel yield prediction. In the subsequent model-evaluation results, this improvement was reflected in lower RMSE and MAE values and enabled a more effective comparison between conventional ML models and physics-informed neural network (PINN). Although this augmentation method has two practical limitations, the generated points do not constitute new experiments, and the digitization process can be time-consuming. It remains highly useful for literature-driven modeling because it preserves experimentally observed trends while substantially improving sample density and model learnability.

### 3.4 Selection of machine learning models

Six main ML models were selected to represent a broad range of modeling complexity and learning behavior: CatBoost, random forest, SVM, neural network, linear regression, and PINNs. Collectively, these models include linear baselines, kernel-based methods, ensemble approaches, and nonlinear neural network models.

#### 3.4.1 CatBoost model

CatBoost is an advanced gradient boosting algorithm designed to provide high predictive accuracy, strong stability, and efficient computation for complex regression and classification tasks. Like other boosting methods, it builds an ensemble of weak learners sequentially, with each new tree improving the errors of the previous ones. The prediction can be expressed as:2
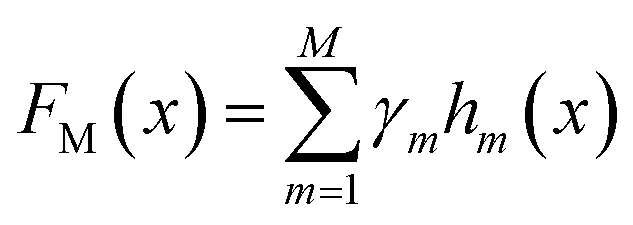
where *h*_*m*_(*x*) is the weak learner at iteration *m*, *γ*_*m*_ is its contribution weight, and *M* is the number of boosting iterations.

The main strength of CatBoost lies in its ability to model nonlinear relationships, variable interactions, and noisy data effectively, without requiring extensive feature engineering. In addition, it introduces ordered boosting and improved handling of feature transformations, which help reduce overfitting and improve generalization on unseen data. Another important advantage is its use of symmetric or oblivious trees, which enhance prediction speed and computational efficiency. Because of these properties, CatBoost is well-suited for applications involving structured datasets with complex dependencies among variables. It is particularly valuable when both predictive performance and robustness are important, and when computational cost must also be considered. Compared with many traditional ML models, CatBoost often provides a strong balance between accuracy, stability, and efficiency, making it a reliable choice as both a benchmark and a practical predictive model.^30^

#### 3.4.2 Linear model

Linear regression was employed as a baseline model to evaluate the extent to which the biodiesel dataset can be described by linear relationships. The model assumes a linear combination of input features:3
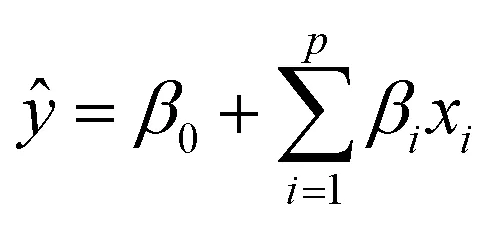
where *β*_0_ is the intercept and *β*_*i*_ is the regression coefficient; they are estimated by minimizing the sum of squared residuals.

Despite its simplicity, interpretability, and computational efficiency, linear regression is limited in its ability to capture nonlinear dependencies and interactions among variables. In complex systems such as biodiesel production, where reaction kinetics and process conditions exhibit nonlinear behavior, LR often underperforms compared to more flexible models. As observed in the results, its relatively poor predictive accuracy confirms the inherently nonlinear nature of the dataset and justifies the use of advanced ML approaches.

#### 3.4.3 Random forest model

RF is an ensemble-based learning method that generates a collection of decision trees from bootstrapped samples of the original dataset. During tree construction, a random subset of predictor variables is considered at each split, promoting variability among individual trees and reducing their dependence on one another. The final prediction is obtained by averaging the outputs of all trees:4
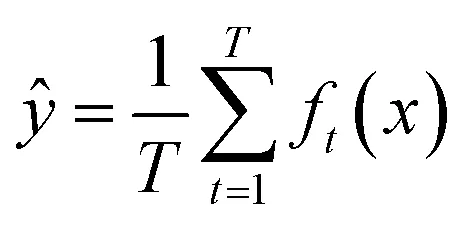
where *T* denotes the total number of trees and *f*_*t*_(*x*) represents the prediction of the *t*-th decision tree. This ensemble mechanism significantly reduces variance compared to a single decision tree and improves generalization performance.

RF is particularly advantageous for modeling complex nonlinear relationships and feature interactions without requiring explicit feature engineering or strict assumptions about data distribution. Additionally, it is robust to noise, outliers, and multicollinearity, which are common in literature-derived biodiesel datasets. Its relatively low sensitivity to hyperparameter tuning and strong performance on small-to-medium datasets make it a practical and reliable benchmark model for nonlinear regression problems in chemical process modeling.

#### 3.4.4 Support vector machine model

Support vector machine, implemented here in its regression form, is a powerful supervised learning method that constructs a function within a predefined tolerance margin, *ε*, while minimizing model complexity.^14^ The optimization problem is formulated as:5
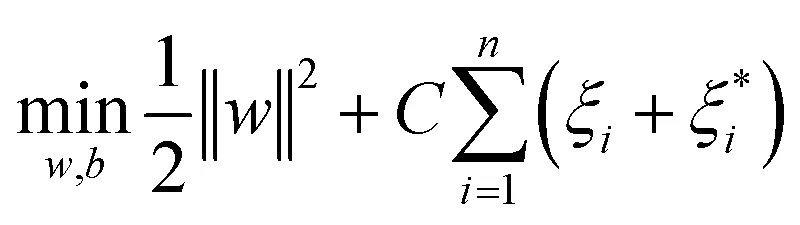
subject to:

6 *y*_*i*_ − (*w* × *ϕ*(*x*_*i*_) + *b*) ≤ *ε* + *ξ*_*i*_

7
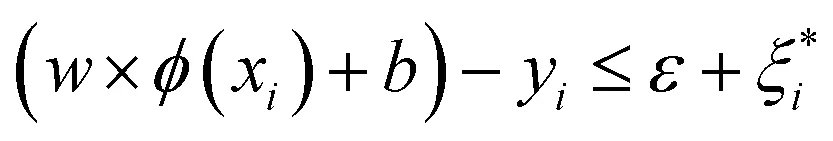
where *C* is the regularization parameter controlling the trade-off between model flatness and tolerance to deviations, *ξ*_*i*_, 
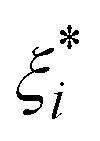
 are slack variables, and *ϕ*(*x*) is a nonlinear mapping induced by a kernel function such as the radial basis function (RBF):

8 *K*(*x*_*i*_,*x*_*j*_) = exp(−*γ*∥*x*_*i*_ − *x*_*j*_∥^2^)

SVM is especially effective for small datasets due to its reliance on a subset of training samples (support vectors), which define the regression function. Its ability to capture nonlinear relationships through kernel transformations makes it well-suited for biodiesel modeling, where process variables exhibit complex interactions. Furthermore, SVM has been widely reported in previous studies for both predictive modeling and hybrid optimization of biodiesel production systems, reinforcing its role as a strong non-tree-based nonlinear benchmark.

#### 3.4.5 Neural network model

The neural network model was implemented as a multilayer perceptron regressor. The proposed model used three hidden layers, together with ReLU activation. This model family was expected to capture complex interactions among catalyst, feedstock, and operating variables. Because neural networks are sensitive to data scaling and encoding, they benefited strongly from the preprocessing and data-augmentation steps.^31^

The neural network model was implemented as a multilayer perceptron (MLP) regressor, consisting of three fully connected hidden layers with 128, 64, and 32 neurons, respectively. Each hidden layer employs the Rectified Linear Unit (ReLU) activation function:

9 ReLU(*x*) = max(0,*x*)

The model approximates a nonlinear mapping between inputs and outputs:

10 *ŷ* = *f*(*x*;*θ*)

where *θ* represents the set of learnable weights and biases optimized through backpropagation using gradient-based methods.

MLPs are universal function approximators that may represent extremely intricate, nonlinear relationships between target variables and input characteristics. In biodiesel production systems, these models can effectively capture intricate interactions among catalyst composition, feedstock properties, and operating conditions. However, neural networks are highly sensitive to input scaling, feature encoding, and dataset size.^20^ Therefore, preprocessing techniques such as normalization and encoding, along with data augmentation strategies, were essential to enhance training stability and predictive performance. The architecture selected in this study balances model capacity and generalization ability, making it suitable for medium-sized experimental datasets.

#### 3.4.6 Physics-informed neural network

Physics-Informed Neural Networks (PINNs) are hybrid modeling frameworks that integrate domain-specific physical laws with data-driven learning. Unlike conventional neural networks that rely purely on empirical data, PINNs embed governing equations such as mass balance, energy balance, or reaction kinetics directly into the training process.^32^ This physics-guided constraint reduces the feasible solution space and improves model behavior, particularly in data-limited or noisy environments.

In biodiesel production modeling, PINNs are particularly useful because they can incorporate reaction mechanisms and kinetic laws, thereby improving interpretability, robustness, and extrapolation capability. Building on this concept, the present study develops a kinetics-guided PINN benchmark that combines an Arrhenius-type reaction rate with a learnable function to capture additional system effects.

In this formulation, the PINN model was formulated to predict biodiesel conversion as a function of reaction time, temperature, and the remaining process and catalyst descriptors:

11 *X̂*(*t*,*T*,*z*) = *f*_*θ*_(*t*,*T*,*z*)

where *t* is the reaction time, *T* is the absolute temperature, and *z* represents the remaining operating, feedstock, and catalyst-related variables. Since the target variable was normalized as conversion, the biodiesel yield was recovered as:

12 *Ŷ* (%) = 100*X̂*

To introduce physical consistency into the learning process, the model is constrained using a reaction-kinetic formulation. Specifically, the conversion dynamics are guided by an Arrhenius-type expression, where temperature-dependent kinetics are combined with a learnable correction term representing complex system effects:13
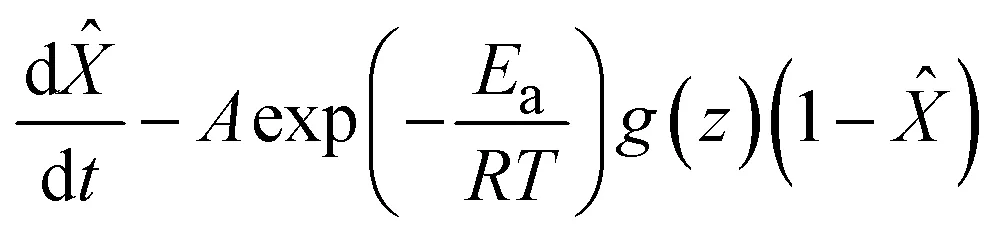
Here, *A* is the pre-exponential factor, *E*_a_ is the activation energy, and *R* is the universal gas constant. The term *g*(*z*) is a learnable function that captures the combined influence of catalyst properties, feedstock characteristics, and operating variables not explicitly represented by time and temperature.

To enforce consistency between the neural prediction and the governing kinetics, a physics residual is defined as the deviation between both expressions:14
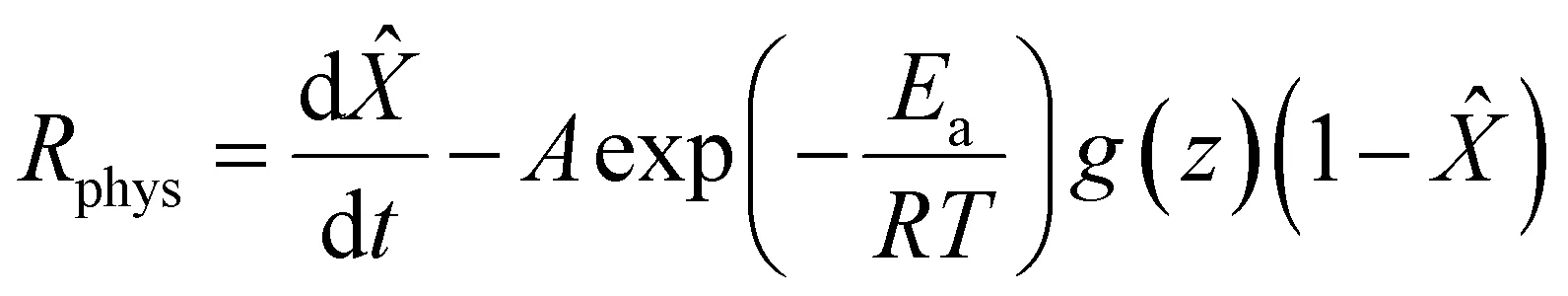


The PINN is trained by minimizing a combined objective function that integrates both data fidelity and physics consistency.

Overall, this formulation enables the model to simultaneously learn from data while being guided by reaction-kinetic structure, ensuring physically consistent predictions while maintaining flexibility to capture nonlinear relationships in biodiesel yield behavior.

### 3.5 Machine learning model development

#### 3.5.1 Model training and cross-validation strategy

**Fig. 4:**
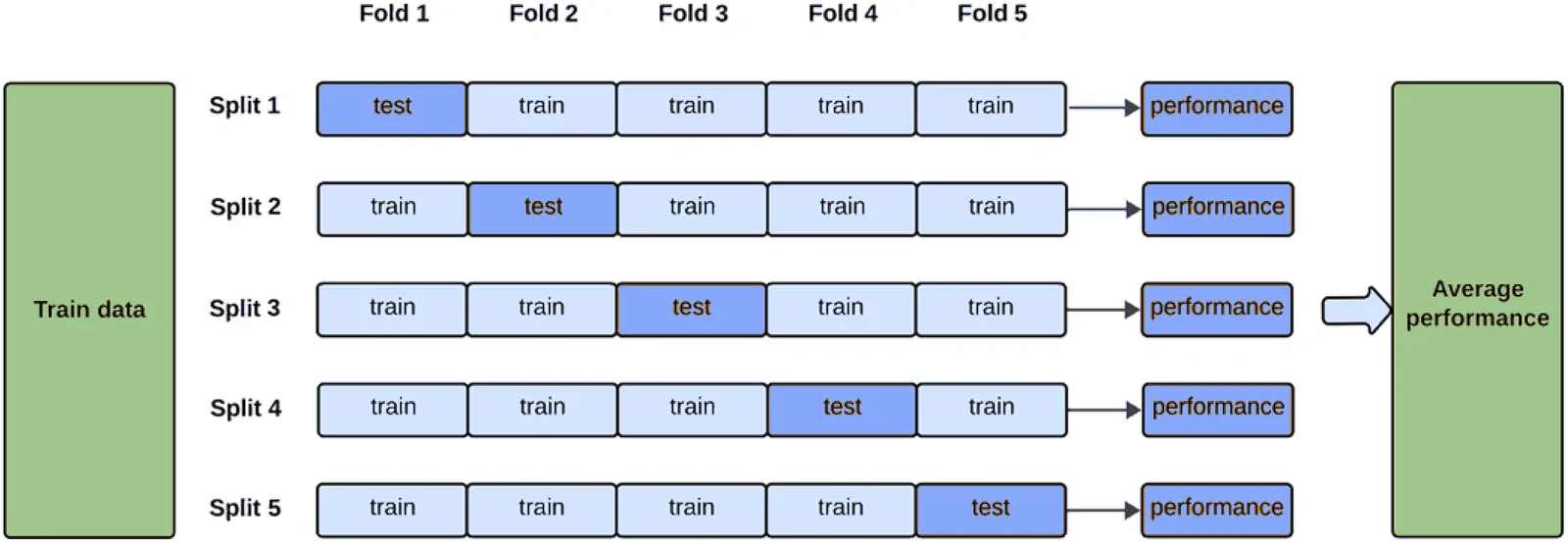
Schematic illustration of 5-fold cross-validation for model evaluation.

The modeling workflow followed a supervised-regression pipeline that began by reading the spreadsheet data, constructing preprocessing steps, training the selected regressor, and evaluating the model through 5-fold cross-validation. This cross-validation strategy improves model reliability by efficiently utilizing the available data, reducing the risk of overfitting, and providing a more robust estimate of generalization performance through averaging across multiple train–test splits.^17^ After cross-validation, each model was re-fitted on the training subset and then used to predict the unseen evaluation subset, as demonstrated in Fig. 4.

To ensure unbiased evaluation, the augmented dataset was split at the study level. Data from multiple studies were used for model development, whereas the remaining studies were reserved exclusively as an unseen holdout set for final model assessment. Cross-validation metrics are reported as the mean values over the folds. The same evaluation metrics were then computed on the holdout set to assess generalization. This protocol is especially important for literature-based data because internal model fit alone can overestimate model reliability when data are heterogeneous.

The workflow also included a comprehensive training and testing strategy implemented through a unified ML pipeline. Multiple regression algorithms were evaluated, including SVM, RF, LR, CatBoost, neural network, and PINNs models, to ensure a robust comparison of model performance. Initially, the dataset was preprocessed by encoding category variables, normalizing numerical features, and addressing missing values. For the neural network model, this preprocessing was implemented using Python-based pipelines with imputation, scaling, and one-hot encoding, followed by training with sklearn's MLPRegressor. For tree-based and boosting models such as RF and CatBoost, appropriate handling of categorical and numerical features was applied, with CatBoost directly leveraging categorical feature support.

Hyperparameters were optimized exclusively on the training dataset using five-fold cross-validation to prevent information leakage and ensure robust model generalization. For each model, the optimal hyperparameter configuration was selected based on the lowest cross-validated RMSE. The finalized hyperparameter settings for all models are summarized in Table 3. These optimal configurations were then used to retrain the models on the full training data. In contrast, the independent holdout dataset was strictly reserved for final performance evaluation and was not involved in any stage of hyperparameter tuning or model selection.

**Table 3:** Tuned hyperparameters and final configurations of predictive models

Model	Hyperparameters	Final selected values
CatBoost	*N* estimators, min samples split, and min samples leaf	200, 2, 1
Random forest	*C*, *γ* (gamma), kernel, and *ε*	100, 0.01, RBF, 0.1
SVM	Iterations, depth, learning rate, and L2 leaf regularization	2000, 8, 0.03, 3
Neural network	Hidden layer sizes, activation, alpha, and initial learning rate	(256, 128, 64, 32), ReLU, 1 × 10^−4^, 1 × 10^−3^
PINNs	Architecture, training, and physics weights	lr = 0.001, layers = 4, neurons=(256, 128, 64, 32), *λ* data = 1, *λ* physics = 0.05

#### 3.5.2 Model loss functions

The ML models used in this work rely on different objective loss functions depending on their learning mechanisms. Linear regression, CatBoost, and the neural network were trained by minimizing squared-error-based losses. For linear regression, this is expressed as the residual sum of squares:15
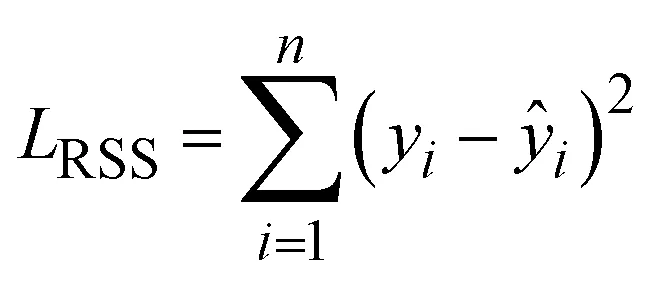
where RSS is the residual sum of squares, *y*_*i*_ is the observed value, *ŷ*_*i*_ is the predicted value, and *n* is the number of samples. For CatBoost and the neural network, the optimization is based on the mean squared error:16
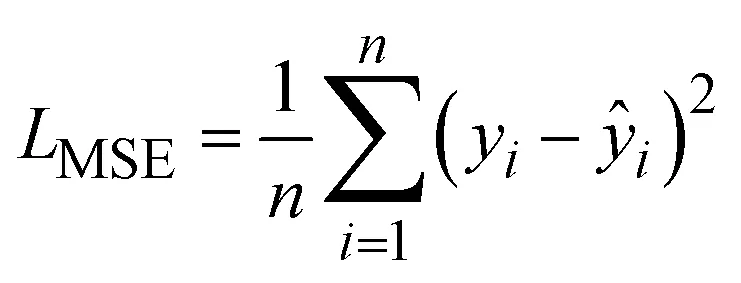
where MSE is the mean squared error, *y*_*i*_ is the true value, *ŷ*_*i*_ is the predicted value, and *n* is the total number of samples. In contrast, the SVM model uses the *ε*-insensitive loss function:

17 *L*_*ε*_(*y*,*ŷ*) = max(0,∣*y* − *ŷ*∣ − *ε*)

where *L*_*ε*_ is the loss value, and *ε* is the error tolerance within which deviations are not penalized.

RF does not employ a single global loss function; instead, it uses a local squared-error criterion at each split to reduce node impurity:18
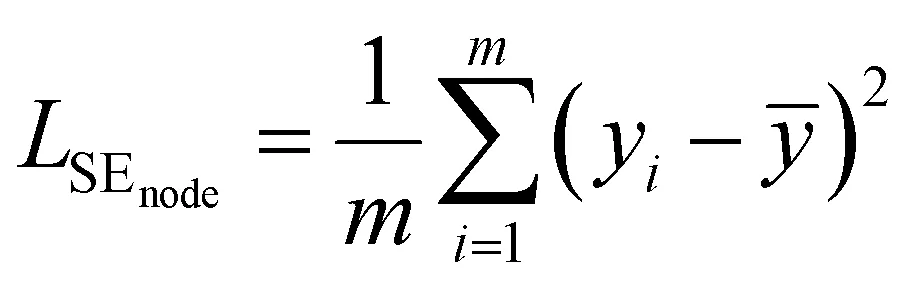
where SE_node_ is the squared error within a tree node, *y*_*i*_ is the target value of sample *i*, *ȳ* is the mean target value in that node, and *m* is the number of samples in the node.

For the PINNs model, the loss function combines a data-fitting term with a physics-based penalty term, so that the model satisfies both the observed data and the governing physical relationships. The total loss is written as:19
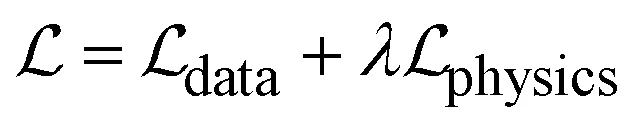


The data-fitting term is defined as:20
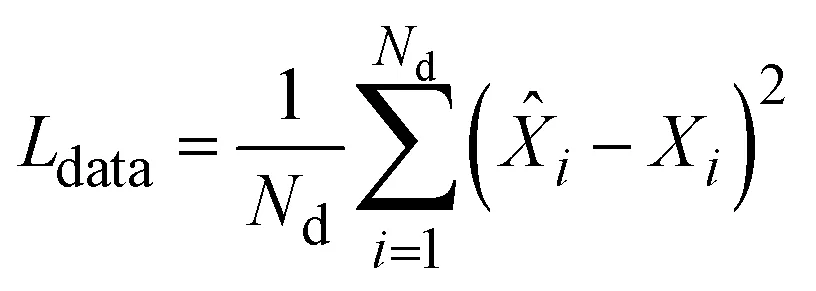
and the physics-based penalty term is expressed as:21
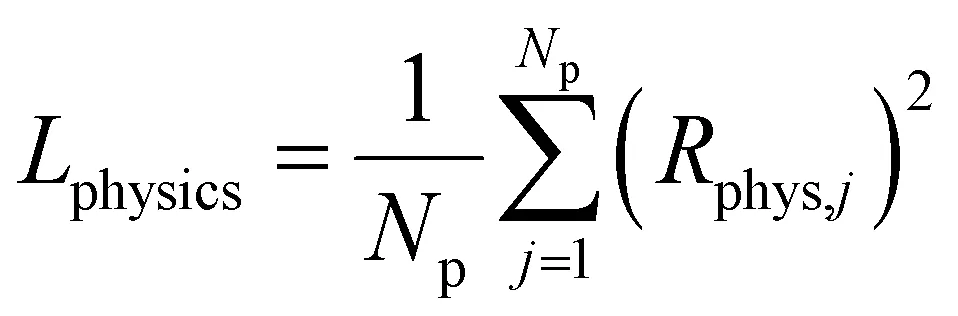
where *L* is the total PINNs loss, *L*_data_ is the data-fitting loss, *L*_physics_ is the physics-based residual loss, *N*_d_ is the number of data samples, *N*_p_ is the number of physics collocation points, *X̂*_*i*_ is the predicted conversion, *X*_*i*_ is the observed conversion, and *R*_phys,*j*_ is the physics residual at the collocation point *j*. The physics residual is obtained from the governing differential operator *N*[·] applied to the neural-network approximation *u*(*x*;*θ*), where *θ* denotes the trainable parameters. The weighting factor *λ* balances data fidelity and physical consistency. Together, these objective functions determine how each model learns the relationship between the selected input variables and biodiesel yield.

### 3.6 Model performance evaluation

Model accuracy was assessed using root mean squared error (RMSE), mean absolute error (MAE), mean absolute percentage error (MAPE), coefficient of determination (*R*^2^), and, where reported, the maximum absolute error. These metrics are defined as follows:22
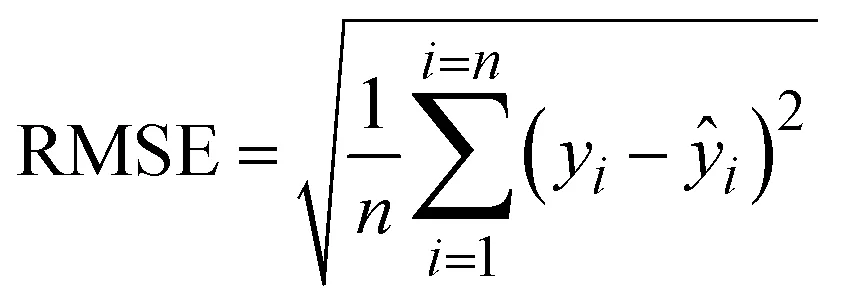
23
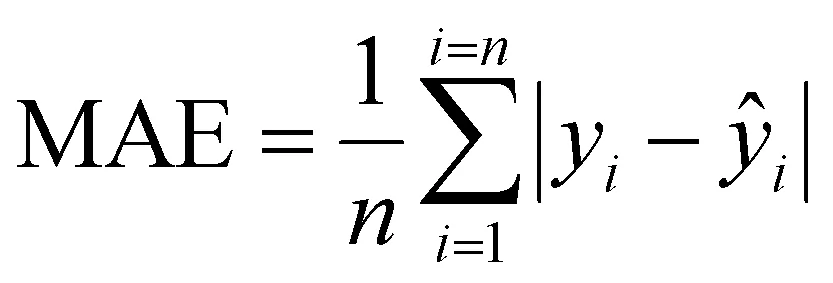
24
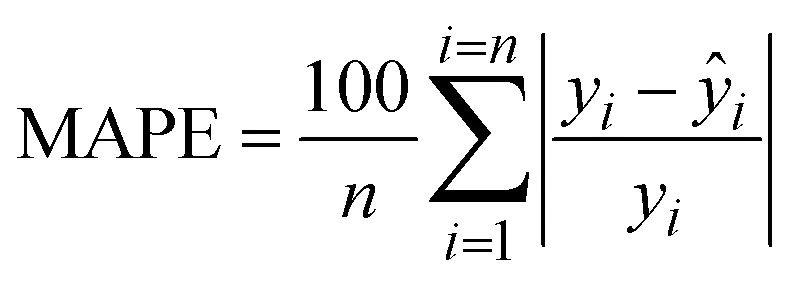
25
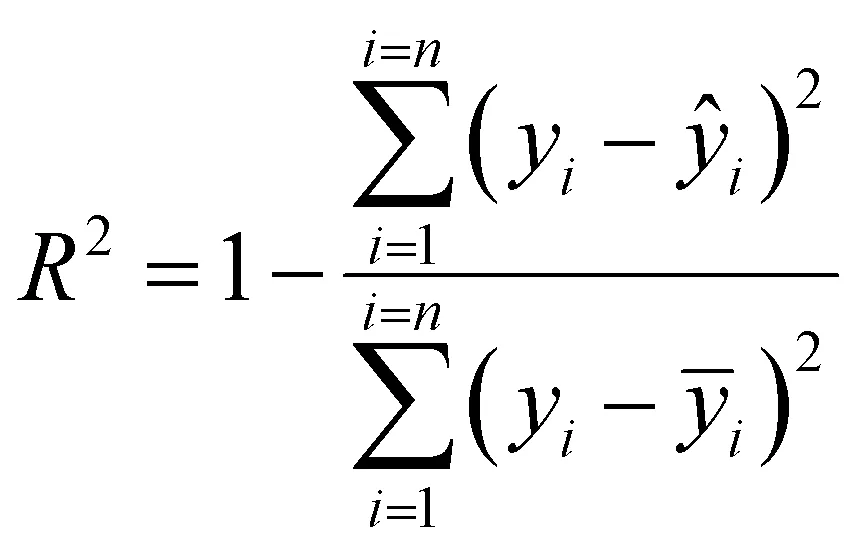
where *y*_*i*_ is the observed value, *ŷ*_*i*_ is the predicted value, *ȳ* is the mean of observed values, and *n* is the number of samples.

RMSE gives greater weight to larger prediction errors and is therefore useful for penalizing poor predictions. MAE provides the average magnitude of the error and is less sensitive to outliers than RMSE. MAPE expresses the error relative to the true value and is useful for comparing accuracy in percentage terms. *R*^2^ measures the extent to which the model explains the variance in the observed yields; values nearer 1 indicate a better match.^20^ The RMSE was calculated in the same unit as the target variable; therefore, since biodiesel yield was expressed as a percentage (%), RMSE values are reported as percentage points (%).

These metrics were applied to the unseen test dataset to offer a thorough evaluation of the accuracy of the model, predictive capability, and overall convergence behavior. By evaluating performance on data that were not used during model training, the reliability and generalization ability of each model could be examined more objectively. The results obtained from applying these metrics to the different ML models are reported and discussed in the Results section.

### 3.7 Feature importance analysis

Feature importance analysis was conducted to move beyond purely predictive performance and to identify the input variables that most strongly influence model predictions. This interpretability technique quantifies the contribution of each feature to the predictive performance, enabling a deeper understanding of the relationships between experimental parameters and biodiesel yield. In this study, it is particularly valuable for determining the influence of key variables such as catalyst amount, reaction temperature, alcohol-to-oil molar ratio, metal loading, and surface area. Such insights are essential for catalyst screening and process optimization, as they link data-driven predictions with chemical understanding.

**Fig. 5:**
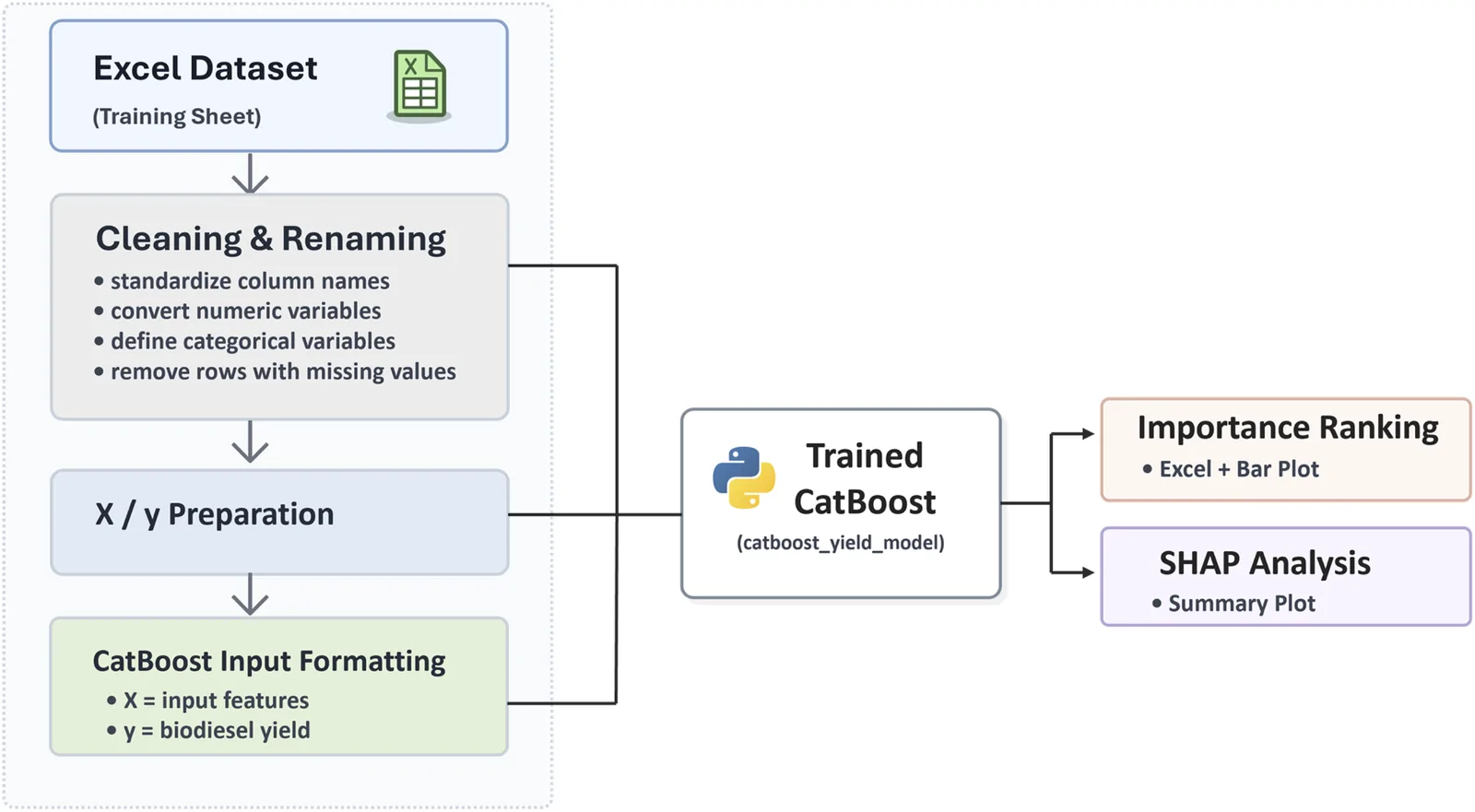
Workflow for CatBoost-based feature importance analysis.

Moreover, feature importance analysis enhances model transparency, helps identify the most influential process parameters, supports validation against established chemical knowledge, and can reduce model complexity by highlighting less relevant variables. To achieve this, two complementary explainability methods were employed: CatBoost feature importance and SHAP analysis. Fig. 5 illustrates the workflow adopted for this explainability analysis, beginning with dataset preprocessing and input preparation, followed by training of the CatBoost model and subsequent interpretation through feature importance ranking and SHAP-based analysis.

#### 3.7.1 CatBoost feature importance

CatBoost feature importance provides a global, dimensionless ranking of variables based on their contribution to reducing prediction error across the ensemble of decision trees.^33^ Although the neural network achieved the highest predictive accuracy, prediction performance and model interpretability represent different objectives. CatBoost was selected for the explainability analysis because it provides robust intrinsic feature-importance measures and supports the exact TreeSHAP algorithm, enabling stable and computationally efficient interpretation of mixed numerical and categorical features. In contrast, explaining neural network predictions typically relies on model-agnostic approximation methods that are computationally more demanding and may exhibit greater variability. Therefore, CatBoost was adopted as the explainable model, while the neural network was retained as the highest-performing predictive model.

For tree-based boosting models, the importance of feature *j* can be expressed as the cumulative reduction in the loss function produced by all splits based on that feature:26
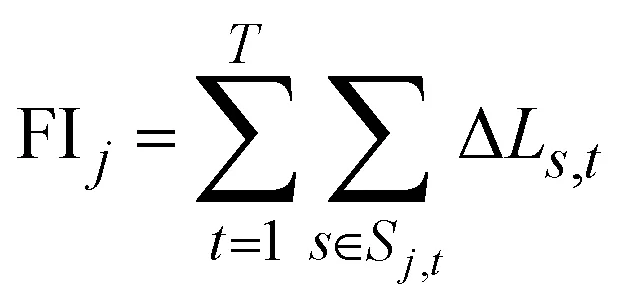
where FI_*j*_ is the importance score of feature *j*, *T* is the number of trees, *S*_*j*,*t*_ is the set of splits using feature *j* in tree *t*, and Δ*L*_*s*,*t*_ is the loss reduction generated by the split *s*. For comparison across variables, the feature-importance scores are normalized as:27
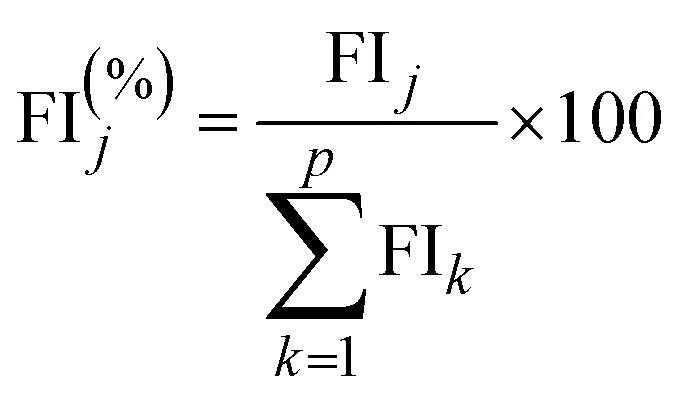
where *p* is the total number of features. This formulation provides a global measure of how strongly each variable contributes to the predictive performance of the CatBoost model.

#### 3.7.2 SHAP analysis

SHAP (Shapley Additive Explanations) was employed as a complementary explainability approach to provide both the magnitude and direction of feature effects. Unlike global importance rankings, SHAP values quantify how each feature contributes to individual predictions by indicating whether it increases or decreases the predicted output relative to the mean prediction. This allows for a more detailed interpretation of model behavior.^34^ Consequently, SHAP analysis complements CatBoost feature importance by not only identifying the most important variables but also revealing how variations in these variables influence biodiesel yield predictions.

For a given sample, the model output is decomposed as:28
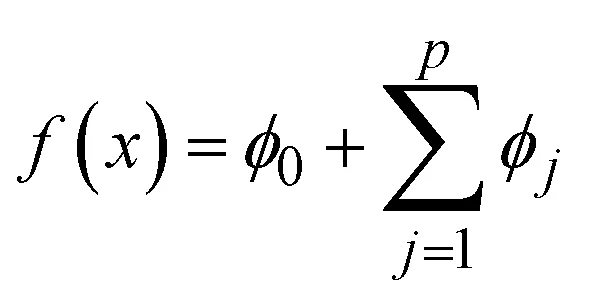
where *f*(*x*) is the model prediction, *ϕ*_0_ is the base value, and *ϕ*_*j*_ is the SHAP contribution of the feature *j*. The SHAP value for the feature *j* is defined as:29
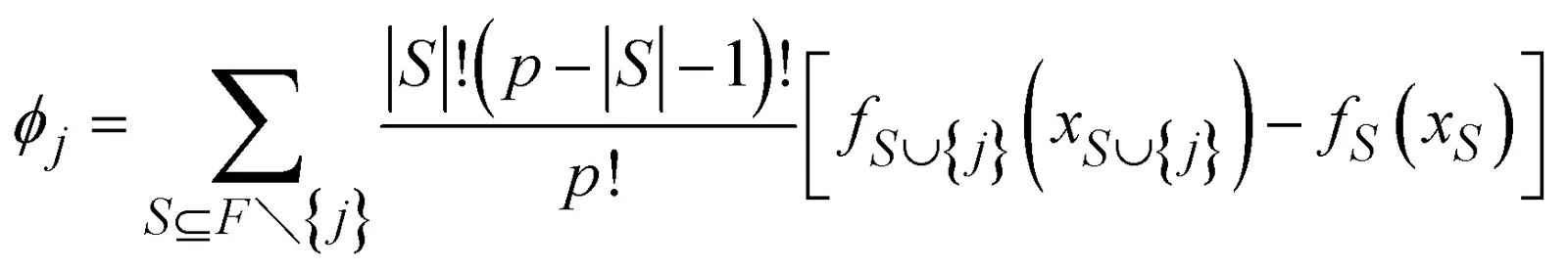
where *F* is the full feature set and *S* denotes a subset of features excluding the feature *j*. Positive SHAP values indicate that a feature increases the predicted biodiesel yield relative to the baseline, whereas negative values indicate that it lowers the prediction. To assess the overall influence of each feature across the full dataset, the global SHAP importance of the feature *j* can be calculated as the mean absolute SHAP value:30
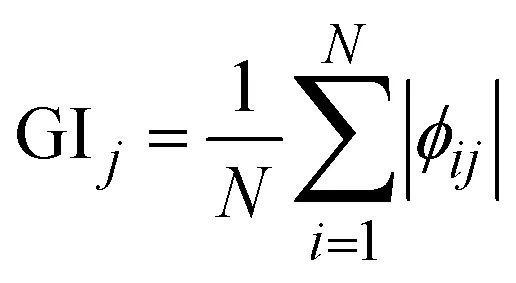
where GI_*j*_ is the global importance of the feature *j*, *N* is the number of samples, and *ϕ*_*ij*_ is the SHAP value of the feature *j* for sample *i*. Thus, SHAP complements CatBoost feature importance by showing not only which variables are most important overall, but also whether high or low values of those variables are associated with higher or lower biodiesel yield.

## 4. Results and explainable machine learning insights

### 4.1 Dataset preparation and augmentation

The final spreadsheet confirms that the augmented modeling dataset is collected from 20 literature case series. After normalization of category labels, the dataset spans 13 feedstock classes, 15 metal classes, and 20 biochar/support-source classes. This diversity is important because it reduces the risk that a model will simply learn a narrow single-feedstock or single-catalyst pattern. The data also cover a wide operating window: acid value ranges from 0.21 to 92.00 mg KOH per g, feedstock FFA from 0.15 to 46.23%, surface area from 1.37 to 812.20 m^2^ g^−1^, alcohol/oil ratio from approximately 2.98 to 24.01, reaction temperature from 20 to 240 °C, reaction time from about 16 to 359 min, catalyst amount from 0.07 to 20 wt%, and yield from 1.00 to 100.00%.

This broad chemical and operational diversity enables the models to learn cross-system catalyst–process interactions rather than a single optimized recipe, reflecting the coupled effects of feedstock properties, catalyst characteristics, and reaction conditions on biodiesel production. However, the right-skewed yield distribution, with a median close to 90%, indicates that high-yield conditions are overrepresented. Therefore, model performance should also be interpreted alongside yield-stratified errors to ensure robust predictive capability across low-, intermediate-, and high-conversion regimes. The descriptive statistics of the principal numerical variables in the augmented dataset are summarized in Table 4.

**Table 4:** Summary statistics for key numerical variables in the augmented dataset

Variable	Minimum	Median	Maximum	Mean
Acid value (mg KOH per g)	0.21	1.19	92.00	5.04
Feedstock FFA (%)	0.15	0.60	46.23	2.56
Metal load (%)	0.00	30.00	350.00	48.85
Surface area (m^2^ g^−1^)	1.37	62.36	812.20	114.50
Alcohol/oil molar ratio	2.98	9.00	24.01	9.63
Reaction temperature (C)	20.00	65.00	240.00	65.40
Reaction time (min)	16.00	150.00	358.60	136.54
Catalyst amount (wt%)	0.07	5.00	20.00	4.48
Mixing intensity (rpm)	400.00	600.00	800.00	614.85
Yield (%)	1.00	89.90	100.00	84.69

Curve digitization increased the numerical resolution of the reported reaction trajectories with an increase of approximately 691%. The value of this procedure is not that it creates new experimental evidence, but that it preserves the curvature of yield-time and yield-condition profiles that would be lost if only the originally tabulated points were retained. Such curvature contains chemically relevant information about rapid initial conversion, approach to equilibrium, catalyst deactivation, or mass-transfer-limited plateaus.

Importantly, the augmentation procedure should not be interpreted as the generation of artificial experimental outcomes. The additional points were directly extracted from experimentally reported profiles and were used only to provide a denser representation of the experimentally observed relationships between reaction conditions and biodiesel yield. Therefore, the purpose of augmentation was to improve the model's ability to capture nonlinear trends and transition regions within the reaction profiles, rather than to artificially increase the amount of independent experimental evidence. The consistency of the extracted trends with the original reported curves ensures that the augmented dataset remains chemically meaningful while providing improved numerical coverage for machine learning model development.

Since adjacent points extracted from the same experimental curve are inherently correlated, they were considered additional representations of the same experimental trends rather than independent experiments. Appropriate grouping during dataset partitioning and cross-validation was maintained to ensure reliable model evaluation and avoid overly optimistic estimates of predictive performance.

**Fig. 6:**
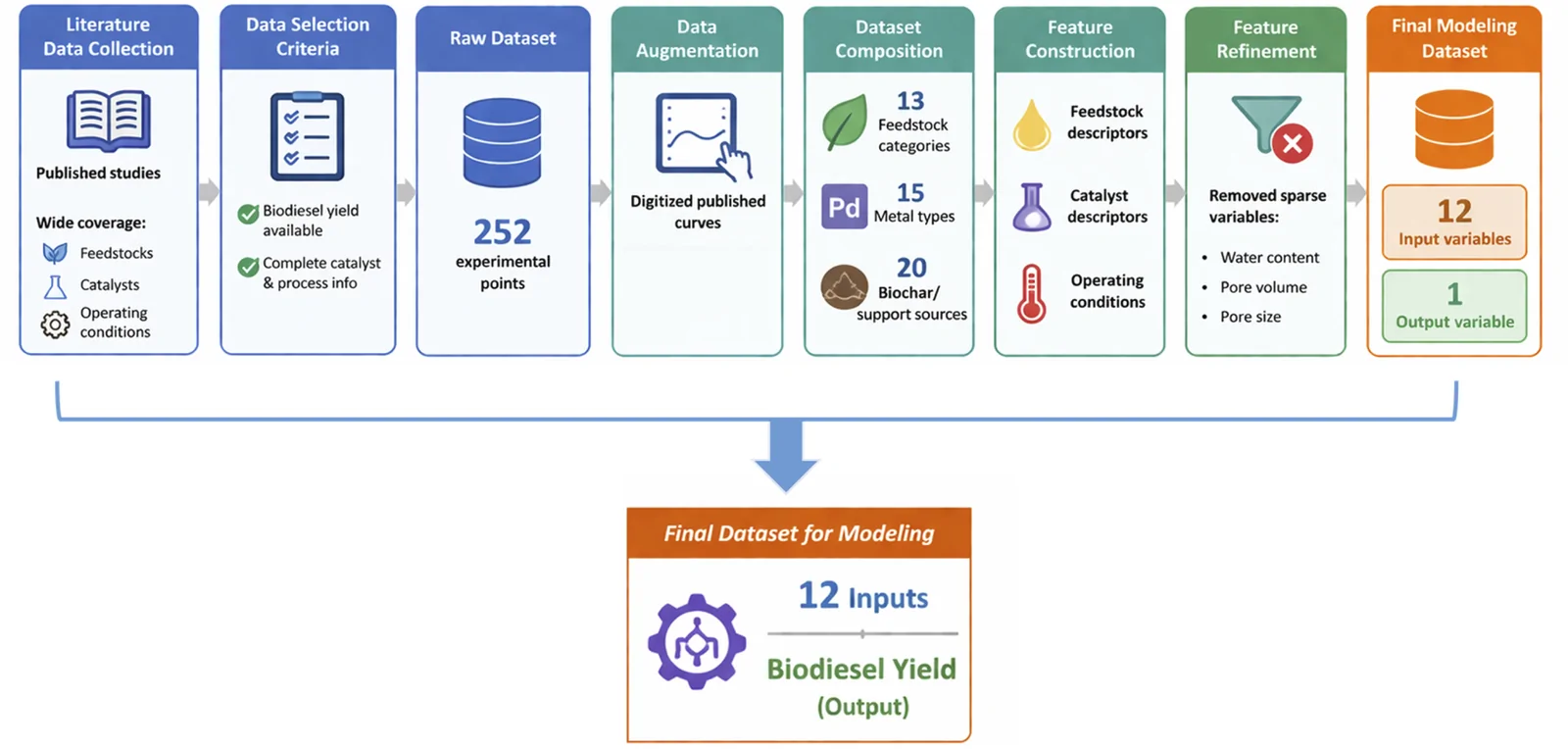
Workflow for dataset preparation, augmentation, and final modeling dataset construction.

The literature-derived nature of the dataset remained evident in the final modeling set. Some potentially informative descriptors were too frequently missing to be retained in the final input space; accordingly, water content, pore volume, and pore size were excluded. These exclusions represent a limitation of the current feature space, as such properties can influence catalyst behavior through effects on hydrolysis, active-site accessibility, liquid uptake, and intraparticle transport. Nevertheless, the data preparation and augmentation strategy yielded a coherent, model-ready dataset with sufficient breadth for comparative ML analysis. As illustrated in Fig. 6, this workflow encompassed literature data collection and screening, data augmentation, feature construction and refinement, and the generation of the final modeling dataset.

### 4.2 Training results of the machine learning models

Hyperparameters were tuned solely on the training data using five-fold cross-validation, ensuring no information leakage. The separate holdout sets were reserved only for final performance assessment and were not used for hyperparameter optimization. Cross-validation performed on the 25-study modeling subset, comprising 1794 data points, demonstrated that nonlinear ensemble methods provided the strongest and most stable predictive performance. This finding provides evidence that biodiesel yield across heterogeneous carbon-supported catalyst systems is governed by nonlinear and conditional relationships rather than simple independent effects of individual variables.

As summarized in Table 5, CatBoost achieved the best overall cross-validation results, with an RMSE of 3.7009%, MAE of 1.7614%, MAPE of 6.13%, and *R*^2^ of 0.9382. These values represent the mean performance averaged over five cross-validation folds. The superior performance of CatBoost arises from its ability to capture complex feature interactions and identify different reaction regimes without assuming a predefined relationship. This highlights that variable effects depend on combined catalyst–process conditions, consistent with multiphase catalytic systems governed by coupled kinetics, phase behavior, mass transfer, and catalyst accessibility.

**Table 5:** Mean five-fold cross-validation performance of machine learning models on the training subset (ranked)

Model	RMSE (%)	MAE (%)	MAPE (%)	*R* ^2^
CatBoost	3.7009 ± 0.8131	1.7614 ± 0.2037	6.13 ± 7.01%	0.9382 ± 0.0200
Random forest	3.9006 ± 0.6920	1.8083 ± 0.1385	6.21 ± 7.04%	0.9316 ± 0.0177
SVM	4.5492 ± 0.6406	2.3614 ± 0.2092	6.93 ± 7.34%	0.9070 ± 0.0175
Neural network	4.8761 ± 0.6885	2.8581 ± 0.3670	7.53 ± 7.67%	0.8930 ± 0.0216
PINNs	8.3816 ± 0.2181	6.3249 ± 0.1267	12.59 ± 3.86%	0.6869 ± 0.0208
Linear regression	13.2768 ± 0.4259	9.7446 ± 0.2403	20.27 ± 4.52%	0.2222 ± 0.0145

To quantify the variability associated with model development and evaluation, the cross-validation results are reported as the mean ± standard deviation (SD) across the five folds (Table 5). The SD values represent the variation in model performance caused by different train–validation partitions and provide a quantitative measure of model stability. The obtained deviations were generally small compared with the corresponding mean values, indicating consistent predictive behavior across different subsets of the dataset. For example, CatBoost showed an RMSE variation of ±0.8131%, MAE variation of ±0.2037%, and *R*^2^ variation of ±0.0200, while random forest exhibited comparable stability with RMSE, MAE, and *R*^2^ deviations of ±0.6920%, ±0.1385%, and ±0.0177, respectively. The remaining models also demonstrated limited fold-to-fold variation, with *R*^2^ standard deviations ranging from ±0.0145 for linear regression to ±0.0208 for PINNs. These results confirm that the reported predictive performance is not strongly affected by the specific train–validation partition and provide evidence of the robustness of the developed modeling framework.

**Fig. 7:**
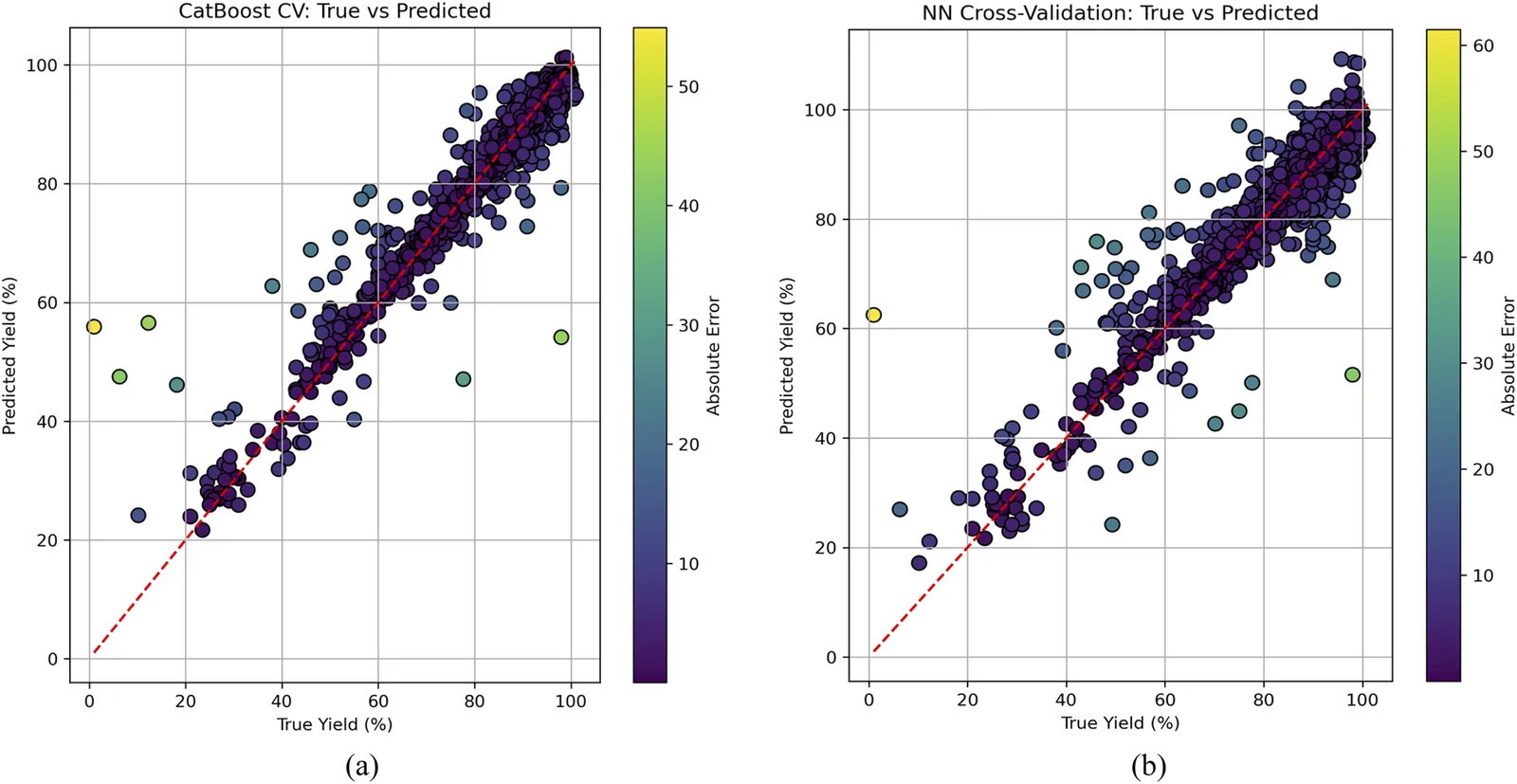
True-*versus*-predicted training results for the developed models: (a) neural network and (b) Catboost.

RF showed comparable performance (RMSE 3.9006%, *R*^2^ 0.9316), confirming the effectiveness of ensemble-based approaches in capturing complex process relationships. This behavior is further illustrated in Fig. 7, where both models exhibit a strong alignment between predicted and actual yields along the ideal parity line. Specifically, Fig. 7(a) corresponds to the CatBoost model, which demonstrates a tighter clustering of data points with fewer high-error deviations, indicating higher prediction consistency and robustness. However, parity plots should be interpreted considering data distribution, as the dominant high-yield region may inflate apparent accuracy; therefore, model reliability should also be assessed across different yield ranges, conditions, and catalyst classes. For clarity, only the training results of these two representative models (CatBoost and neural network) are presented here, while the corresponding results for the remaining models are provided in the SI (Fig. S1).

The SVM model also exhibited strong predictive capability, although slightly inferior to the ensemble models. The neural network model demonstrated competitive performance; however, it yielded a lower cross-validation *R*^2^ value (0.8930), suggesting comparatively reduced stability during training. This lower stability may arise from the sensitivity of neural networks to sample distribution, categorical feature representation, and variations in catalyst composition. As shown in Fig. 7(b), the neural network predictions display a wider dispersion around the parity line, with several noticeable outliers, reflecting higher variability in capturing complex nonlinear relationships. These deviations may also provide scientific insights, as they could indicate catalyst–feedstock systems influenced by unmeasured descriptors, such as active-site characteristics, pore accessibility, water content, or catalyst preparation effects, rather than representing simple model failure.

In contrast, linear regression performed significantly worse than all nonlinear models, with a substantially higher error (RMSE 13.2768%) and a very low *R*^2^ (0.2222). The results confirm that biodiesel yield is governed by nonlinear and conditional relationships, where the effects of temperature, catalyst loading, and acidity depend on catalyst and feedstock characteristics. Linear models are therefore insufficient to represent such complex interactions. The weaker PINN performance likely results from simplified physical constraints that do not fully capture diverse kinetics, adsorption behavior, and mass-transfer effects across heterogeneous catalysts. More comprehensive physicochemical descriptors and system-specific constraints are required to improve physics-informed learning for complex catalytic datasets.

**Fig. 8:**
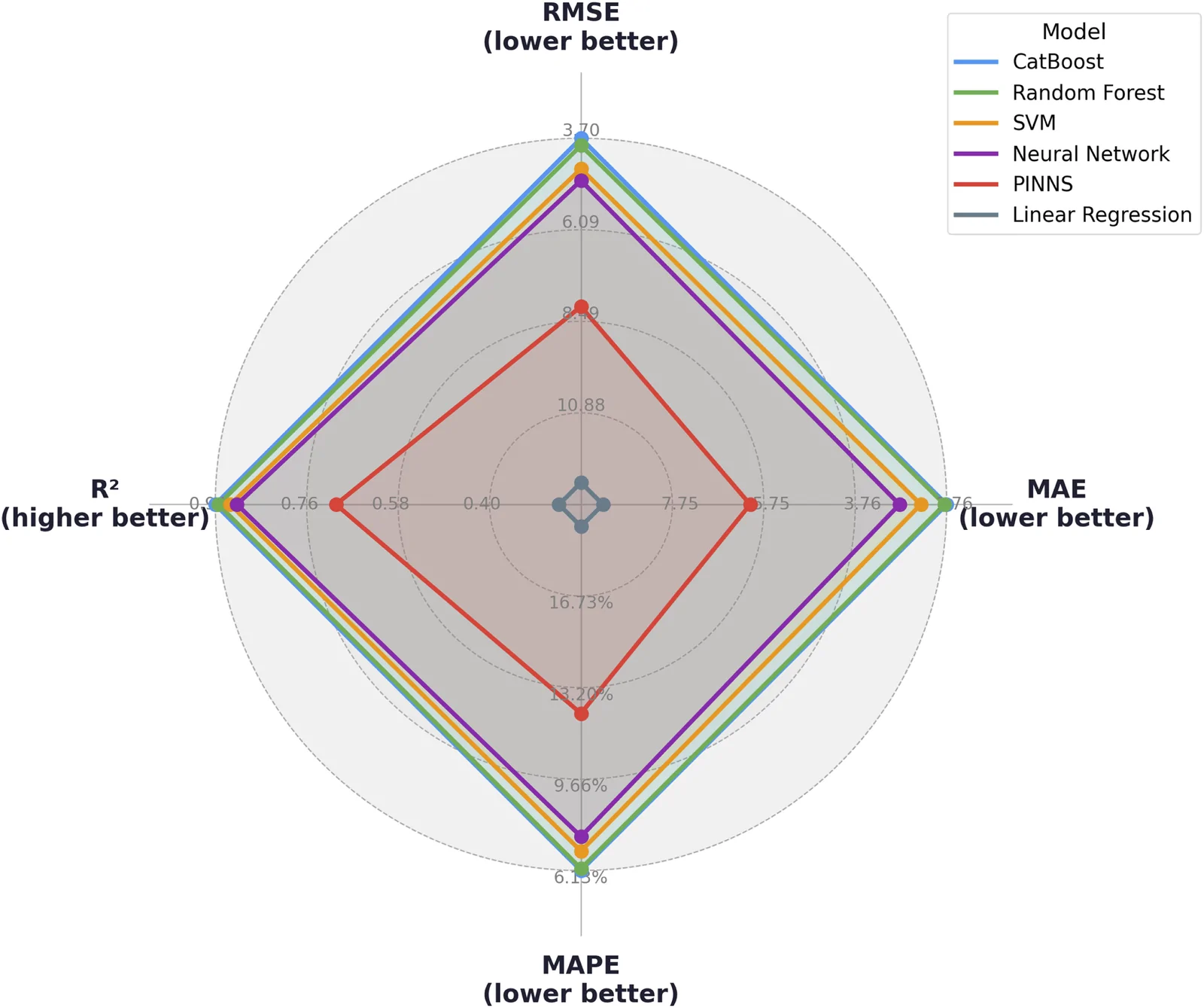
Radar chart comparing cross-validation performance of machine learning models.

The radar chart in Fig. 8 provides a compact visual comparison of the cross-validation performance of all models across RMSE, MAE, MAPE, and *R*^2^. The main implication of this comparison is the separation between model classes, reflecting their different abilities to capture nonlinear catalyst–process relationships. Consistent with the numerical results, CatBoost and random forest occupy the most favorable region, while SVM and the neural network remain competitive but slightly inferior. This performance hierarchy indicates that tree-based ensembles better capture conditional interactions among catalyst properties, feedstock characteristics, and reaction conditions. In contrast, PINNs and especially linear regression are located closer to the center, indicating weaker overall predictive performance. The consistent ranking across multiple metrics confirms that model superiority is not determined by a single evaluation criterion.

### 4.3 Performance evaluation and experimental validation

#### 4.3.1 Assessment of machine learning models

Assessment on the unseen holdout dataset, which included two independent studies totaling 200 data points, further reinforced the consistently superior predictive performance of the nonlinear models. This evaluation provides a more rigorous test of cross-study transferability by assessing whether learned relationships can be applied to unseen catalyst–feedstock systems. As presented in Table 6, the neural network achieved the best overall predictive accuracy, with an RMSE of 3.2724%, MAE of 1.6430%, MAPE of 2.31%, and *R*^2^ of 0.9574.

**Table 6:** Evaluation performance on unseen dataset (ranked)

Model	RMSE (%)	MAE (%)	MAPE (%)	*R* ^2^
Neural network	3.2724	1.6430	2.31%	0.9574
CatBoost	3.4519	1.5506	2.84%	0.9526
SVM	3.7240	1.9844	2.71%	0.9448
Random forest	3.8466	1.7387	3.03%	0.9411
PINNs	8.4850	6.3919	8.82%	0.7133
Linear regression	13.7967	10.2582	16.54%	0.2420

CatBoost closely followed, yielding comparable performance (RMSE 3.4519%, MAE 1.5506%, MAPE 2.84%, *R*^2^ 0.9526), while SVM and RF also maintained high predictive accuracy, each achieving *R*^2^ values above 0.94. The consistent nonlinear model performance indicates that the retained descriptors capture key drivers of biodiesel yield across studies. However, the limited holdout size supports transferability only to the evaluated systems, not universal catalyst-space generalization.

**Fig. 9:**
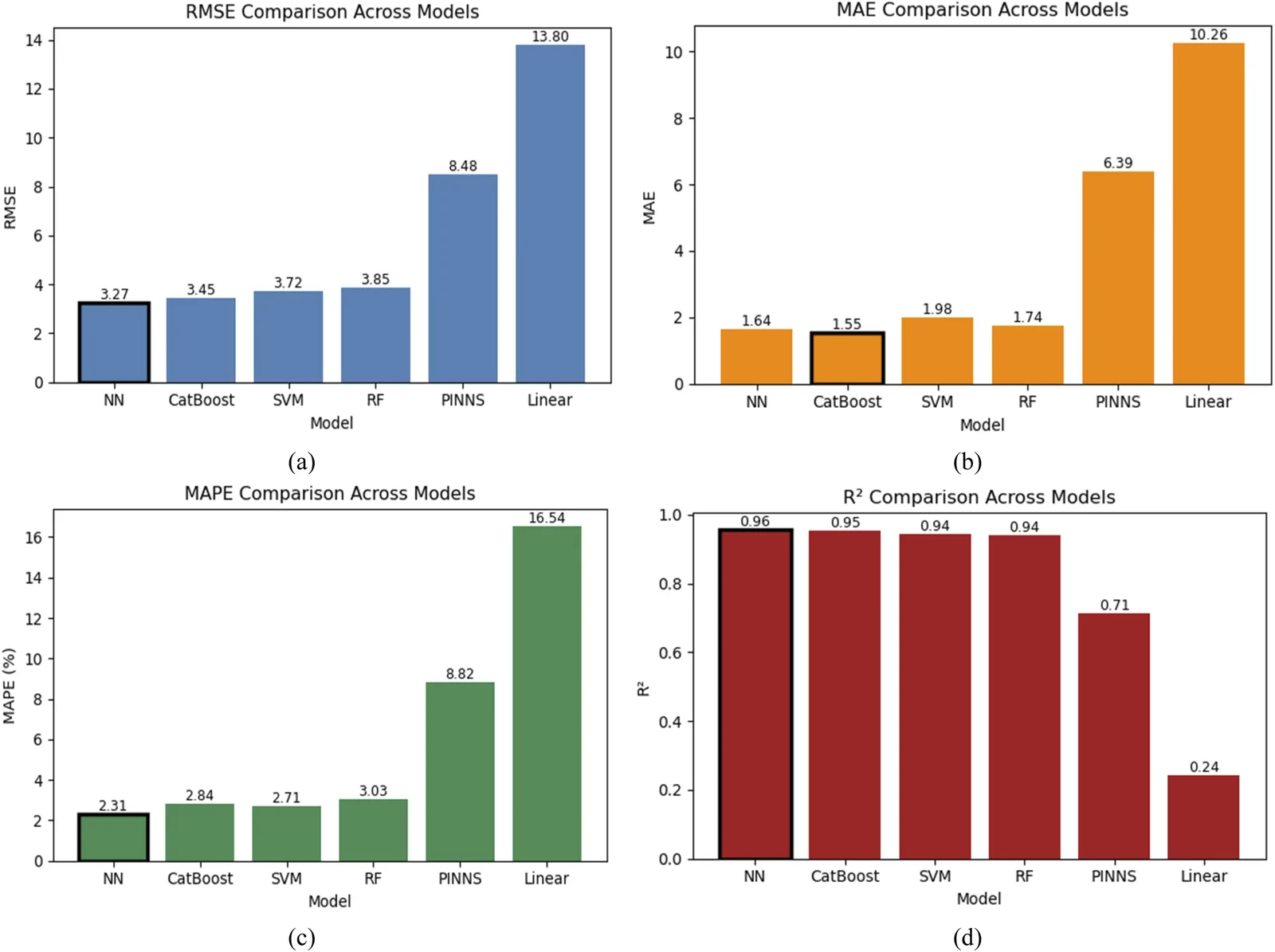
Performance of machine learning models across multiple evaluation metrics: (a) RMSE, (b) MAE, (c) MAPE, and (d) *R*^2^.

Fig. 9 presents a comparison of performance metrics across all the evaluated models considering the unseen dataset, including RMSE, MAE, MAPE, and *R*^2^, shown in subfigures (a), (b), (c), and (d), respectively. These results highlight that model superiority depends on the selected evaluation criterion rather than a single universal metric. As illustrated in Fig. 9(a), the neural network model achieves the lowest RMSE of 3.27%. This indicates stronger control of larger prediction deviations across the holdout dataset.

In Fig. 9(b), CatBoost demonstrates the best performance in terms of MAE, with a value of 1.55%. Fig. 9(c) shows that the neural network model attains the lowest MAPE of 2.31%. Finally, Fig. 9(d) indicates that the neural network model achieves the highest *R*^2^ value of 0.96. Overall, the complementary strengths of neural network and CatBoost suggest that model selection should consider the intended application and error priorities.

In contrast, linear regression exhibited a substantial deterioration in predictive performance, with significantly higher errors and a low *R*^2^ of 0.2420. The consistent underperformance across cross-validation and holdout evaluation confirms that biodiesel yield involves nonlinear effects, including thresholds, saturation, and catalyst–feedstock interactions beyond linear representation. This further confirms that the relationship between process variables and biodiesel yield is inherently nonlinear and cannot be effectively modeled using linear approaches.

The PINN benchmark, although incorporating physical constraints, remained notably less accurate than the data-driven ML models, with higher prediction errors and lower explanatory power. The intermediate PINN performance indicates partial physical relevance but highlights the need for catalyst-specific kinetics and transport descriptors to improve physics-informed models for heterogeneous catalytic systems.

Two key observations emerge from these results. First, dataset augmentation enhanced neural network performance by enabling better learning of complex nonlinear interactions. Second, CatBoost achieved comparable accuracy with greater interpretability and robustness for mixed numerical–categorical inputs. Therefore, CatBoost was selected for subsequent feature-importance and SHAP analyses, providing a balance between predictive capability and explainability.

**Fig. 10:**
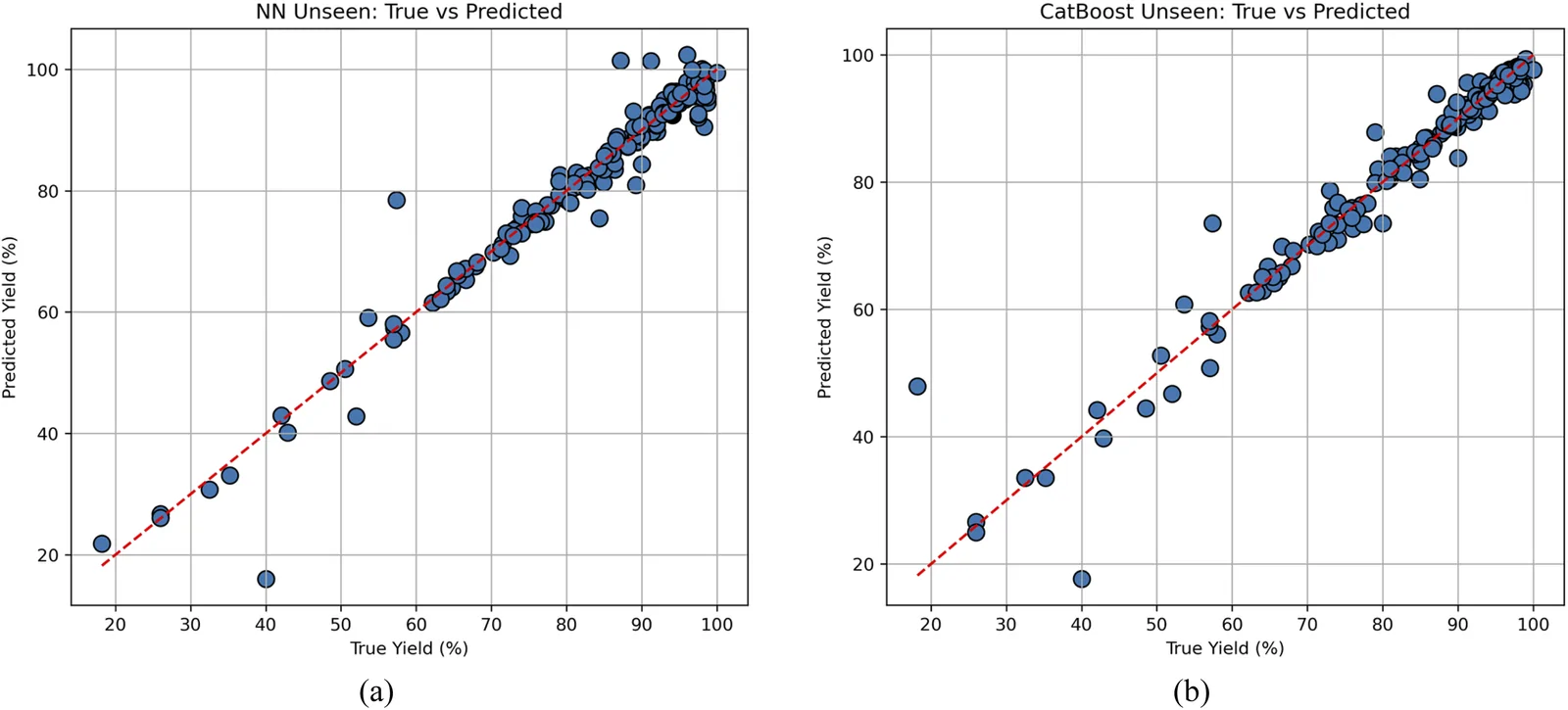
Comparison of predicted *versus* actual yields on the unseen dataset for the developed models: (a) neural network and (b) CatBoost.

Fig. 10 illustrates the comparison between predicted and actual yields on the unseen dataset for two representative models: (a) neural network and (b) CatBoost. These models are highlighted due to their superior predictive performance among the evaluated approaches. The corresponding predicted *versus* actual yield plots for the remaining models are provided in Fig. S2 in the SI Section. The parity plots demonstrate strong agreement between predicted and actual yields; however, deviations from the ideal line provide additional insight into model limitations under different reaction regimes.

Consistent with these findings, the neural network and CatBoost models achieved the highest *R*^2^ values among all evaluated models, with values of 0.9574 and 0.9526, respectively, further confirming their superior ability to capture the variability in the data. These high *R*^2^ values indicate strong variance capture within the evaluated domain, but should be interpreted considering the bounded yield range and the dominance of high-yield samples in the dataset.

**Fig. 11:**
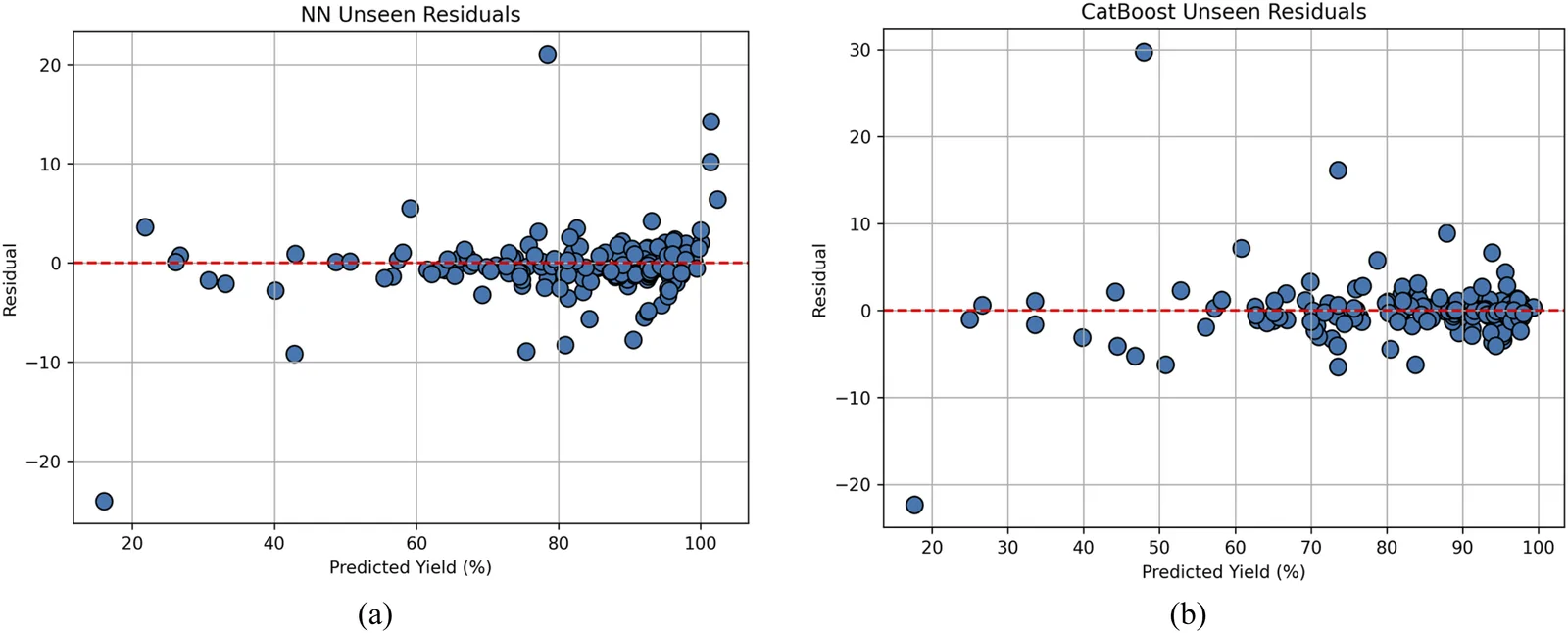
Residual distribution of prediction errors on the unseen dataset for the developed models: (a) neural network and (b) CatBoost.

Moreover, the residual distribution of prediction errors on the unseen dataset for the two best-performing models is shown in Fig. 11. These models are the neural network and CatBoost, which achieved the lowest RMSE values of 3.2724% and 3.4519%, respectively. Residual analysis further evaluates model reliability by identifying systematic errors; residuals centered around zero indicate effective learning, while deviations may reveal unresolved reaction regimes or missing descriptors. The residual distributions for the remaining models are provided in Fig. S3. Differences in residual patterns also explain the balance between RMSE and MAE performance among models.

**Fig. 12:**
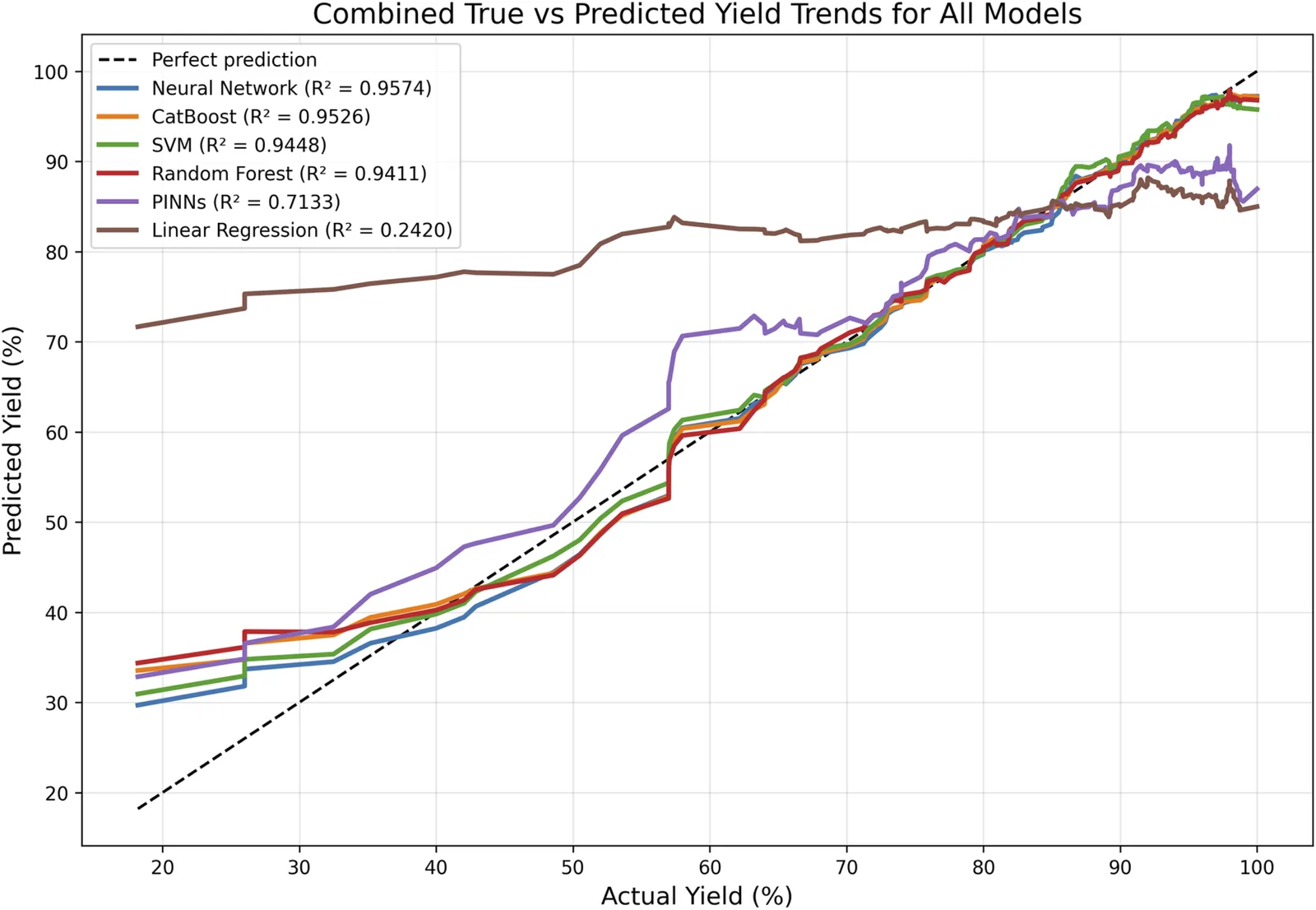
Comparison of true and predicted biodiesel yield trends for all models.

The combined true–predicted yield trends for all models on the unseen testing dataset are represented in Fig. 12. The neural network, CatBoost, SVM, and RF follow the perfect-prediction line closely, indicating strong predictive accuracy and good generalization. The consistent performance of nonlinear models suggests their ability to capture different catalyst–feedstock operating regimes rather than relying on fixed variable effects. The neural network shows the best overall performance, consistent with its lowest RMSE (3.2724%), lowest MAPE (2.31%), and highest *R*^2^ (0.9574), while CatBoost achieves the lowest MAE (1.5506%) and performs comparably well. SVM and RF also show strong agreement with the ideal trend, though with slightly larger deviations.

By contrast, the PINN benchmark departs more noticeably from the ideal line, especially in the intermediate yield range, and linear regression shows the weakest agreement overall, with substantial systematic deviation and the lowest *R*^2^ (0.2420). The PINN deviations suggest limitations in representing mixed kinetic and transport effects using a uniform physical constraint, while linear regression cannot account for regime-dependent variable interactions. These results confirm the superior ability of nonlinear ML models to predict unseen biodiesel-yield data.

#### 4.3.2 Experimental validation

To further assess model robustness, an additional independent experimental dataset reported in the literature was used for external validation.^35^ These datasets were not included in model training or holdout selection and therefore provide a truly independent evaluation of the model's generalization capability.

The selected study investigated biodiesel synthesis using a solid carbon catalyst derived from pyrolytic pomelo peel biomass waste. The catalyst was prepared *via* KOH activation followed by K_2_CO_3_ impregnation and calcination at 600 °C. The optimized catalyst (25 wt% K_2_CO_3_) achieved high biodiesel yield under mild reaction conditions (65 °C, 2.5 h, 8 : 1 methanol-to-oil ratio, and 6 wt% catalyst loading), attributed to enhanced surface properties and strong basic sites.

The study was selected because it offers a coherent series of yields measured for one distinct biomass-derived catalyst under controlled changes in operating conditions. It therefore tests whether the model can transfer learned process–catalyst relationships rather than memorize a specific literature curve. The validation remains local to the descriptor domain represented in training; it does not test extrapolation to fundamentally different catalytic mechanisms, such as enzymatic systems, homogeneous catalysts, supercritical alcohol, or non-carbon supports.

The predictive performance of the proposed models on this independent dataset is summarized in Table 7. The neural network and CatBoost models were selected for external validation as they demonstrated the best overall performance during the model evaluation stage. The neural network model achieved MAE, RMSE, and *R*^2^ values of 2.206%, 2.544%, and 0.955, respectively, while the CatBoost model yielded comparable values of 2.199%, 2.721%, and 0.949. Both models therefore exhibit consistently high predictive accuracy with closely matched performance levels. The comparable performance of two structurally different models supports the robustness of the learned relationships and reduces the likelihood of model-specific bias. Moreover, the models successfully capture both high-yield and incomplete-conversion conditions, indicating applicability beyond optimized reaction points.

**Table 7:** Comparison of actual and predicted biodiesel yields for the independent validation dataset using the neural network and CatBoost models

Data point	Actual yield (%)	Neural network		CatBoost	
		Prediction (%)	Absolute error (%)	Prediction (%)	Absolute error (%)
1	98.00	95.62	2.38	98.56	0.56
2	92.00	87.72	4.28	92.61	0.61
3	89.00	88.10	0.90	92.38	3.38
4	95.00	98.17	3.17	95.53	0.53
5	60.00	64.23	4.23	65.20	5.20
6	66.30	68.62	2.32	70.02	3.72
7	72.70	73.15	0.45	75.92	3.22
8	79.00	77.88	1.12	80.38	1.38
9	83.70	82.11	1.59	83.18	0.52
10	88.30	86.69	1.61	85.42	2.88
MAE (%)		2.206		2.199	
MAPE (%)		2.775		2.991	
RMSE (%)		2.544		2.721	
*R* ^2^ (%)		0.955		0.949	

**Fig. 13:**
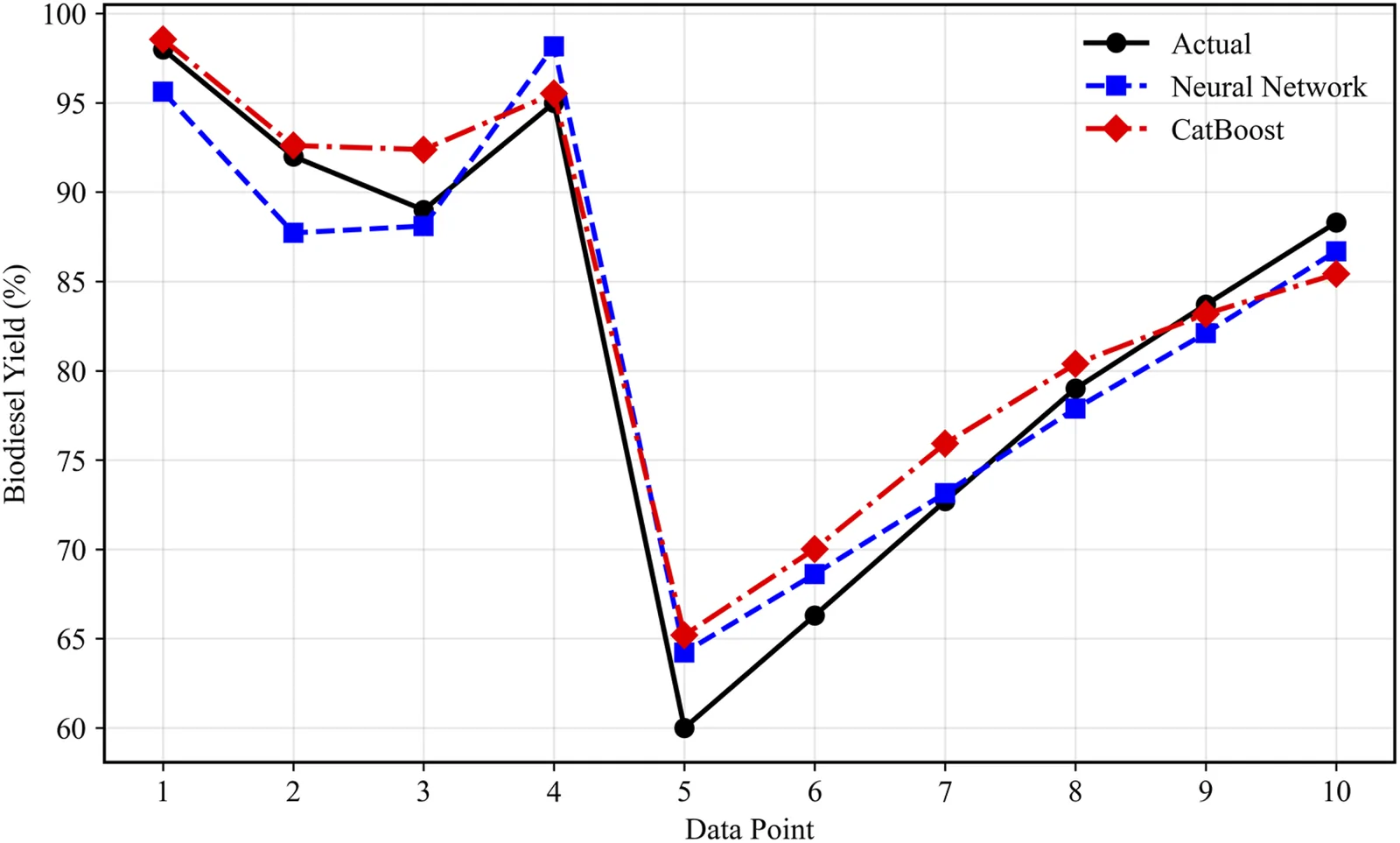
Comparison of experimental and predicted biodiesel yields for independent validation dataset.

A detailed point-by-point comparison between actual and predicted biodiesel yields is presented in Table 7, highlighting the absolute error distribution for both models. The results indicate that CatBoost provides slightly closer predictions in several high-yield cases, while the NN model performs more consistently across the full range of values. The complementary behavior of both models reflects their different learning strategies, where CatBoost captures local response patterns while the neural network provides smoother prediction across the reaction space. Furthermore, the agreement between predicted and experimental biodiesel yields is visually illustrated in Fig. 13, where both models closely follow the experimental trend line with minimal deviation, confirming their strong generalization capability on unseen experimental data.

### 4.4 Explainable ML interpretation, mechanistic insights, and applicability

#### 4.4.1 Results of feature importance and SHAP analysis

**Fig. 14:**
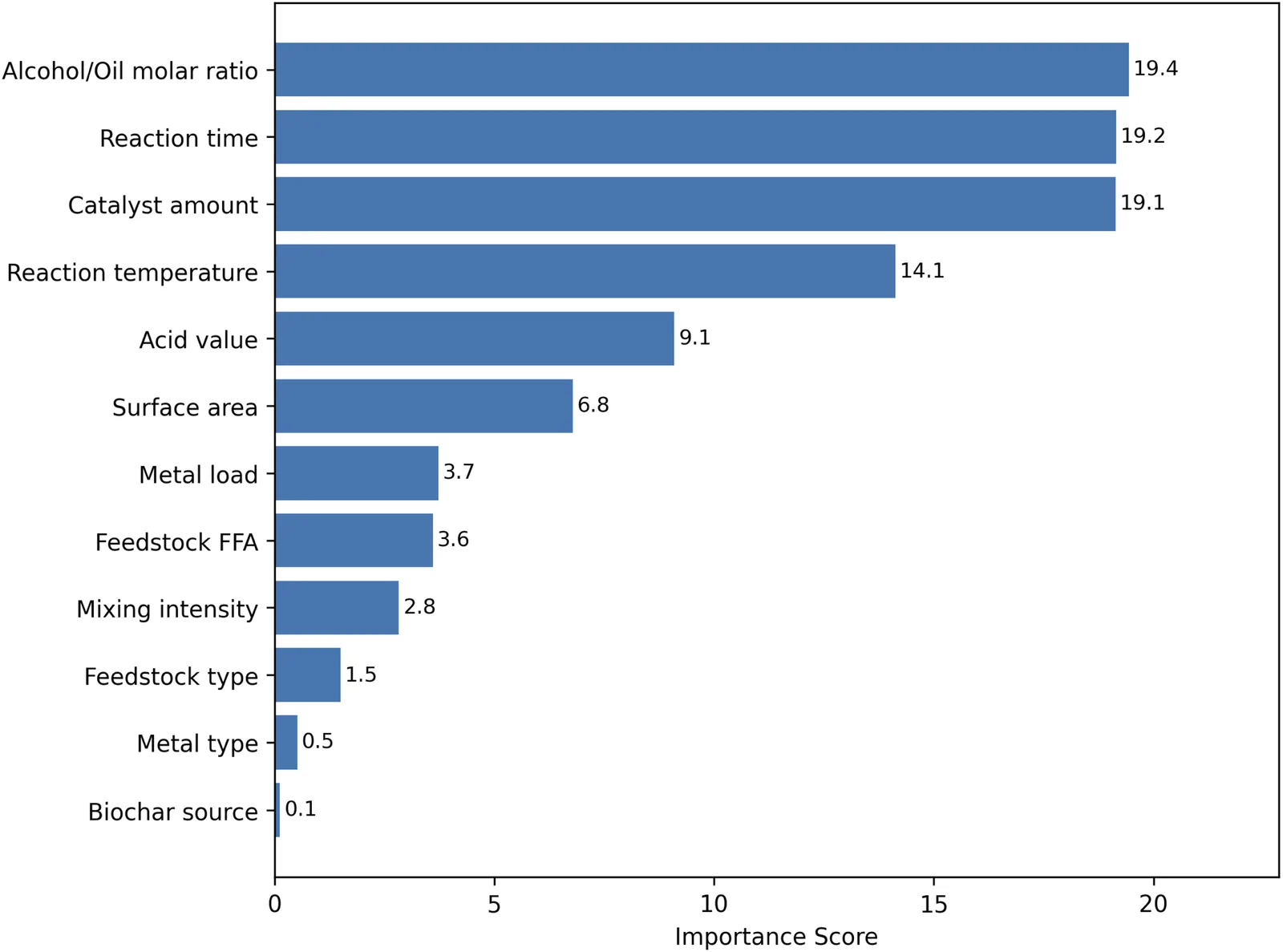
CatBoost global feature-importance histogram analysis.

CatBoost produced a consistent global message: biodiesel-yield prediction in the present dataset is governed primarily by catalyst amount, alcohol-to-oil ratio, reaction time, acid value, and reaction temperature, as described by the histogram analysis in Fig. 14. The statistically similar importance of alcohol-to-oil ratio, reaction time, and catalyst amount suggests that yield is controlled by coupled effects of reactant availability, active-site accessibility, and reaction progression rather than a single dominant factor. Based on the CatBoost importance score calculation, alcohol/oil ratio (19.4), reaction time (19.2), and catalyst amount (19.1) formed a practically tied top tier, followed by reaction temperature (14.1) and acid value (9.1).

The feature-importance analysis confirmed the same five variables as the dominant group, even though the exact ordering of the top three is extremely close. These variables influence biodiesel formation through interconnected effects on reaction kinetics, phase behavior, catalyst accessibility, and reactant conversion. Variables such as surface area, metal loading, feedstock FFA, and mixing intensity contributed smaller but still measurable effects, whereas the categorical variables feedstock type, metal type, and biochar source had relatively modest global scores. The lower importance of catalyst descriptors does not indicate limited chemical relevance but may reflect incomplete reporting of intrinsic properties, such as active-site density, strength, dispersion, and surface functionality.

**Fig. 15:**
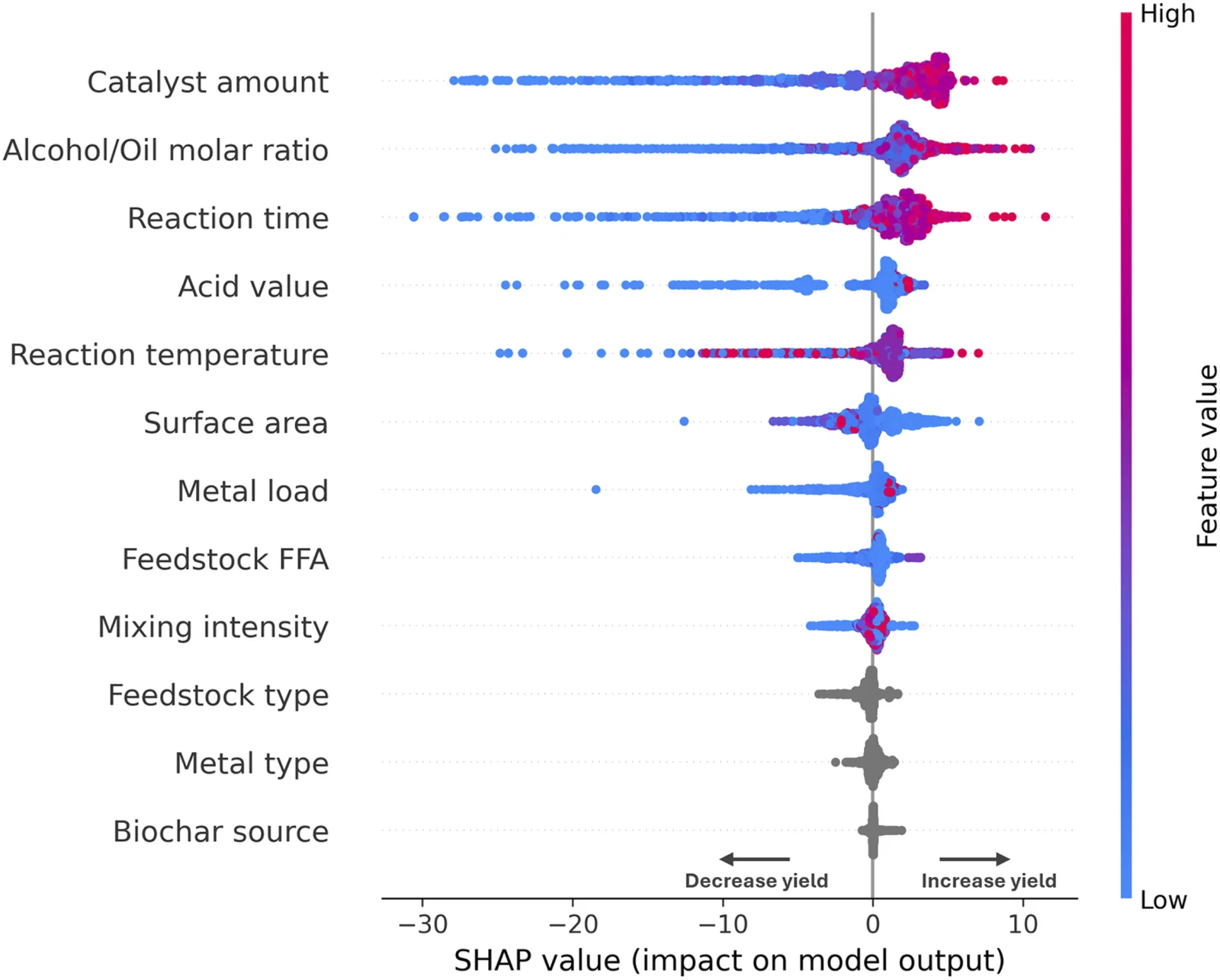
SHAP summary plot of feature importance for biodiesel yield prediction.

The SHAP analysis provided directional insight into the feature-importance ranking, as shown in Fig. 15 and summarized in Table 8, which was derived directly from the computed SHAP values. Each dot in the SHAP scatter plot represents a single sample; the *x*-axis displays the SHAP value; the color denotes the feature value, with red denoting high values and blue denoting low values. A higher anticipated biodiesel yield is indicated by positive SHAP values, whereas a lower yield is indicated by negative values. Unlike simple feature ranking, SHAP values reveal conditional effects, showing how variable contributions change with catalyst, feedstock, and operating conditions. The distribution patterns also highlight potential feature interactions and correlations relevant to mechanistic interpretation.

**Table 8:** SHAP-based interpretation of the major variables affecting predicted biodiesel yield

Feature	Global importance	SHAP-based interpretation
Catalyst amount	Very high	Higher values generally increase predicted yield
Alcohol/oil molar ratio	Very high	Higher values generally increase predicted yield
Reaction time	Very high	Longer reaction times generally increase predicted yield
Acid value	High	Higher acid value generally decreases predicted yield
Reaction temperature	High	Higher values generally increase predicted yield
Surface area	Moderate	Slight net negative effect on predicted yield
Metal loading	Low-moderate	Generally, decreases predicted yield in the fitted model
Feedstock FFA	Low-moderate	Higher values generally increase predicted yield
Mixing intensity	Low	Slight net negative effect on predicted yield
Feedstock/metal type/biochar source	Contextual	These variables influence predicted yield through case-specific material effects

As shown in Fig. 15, catalyst amount, alcohol-to-oil molar ratio, and reaction time were the most influential variables, followed by acid value and reaction temperature. Higher catalyst amount, alcohol-to-oil ratio, reaction time, and reaction temperature were generally associated with increased predicted yield, while higher acid value tended to reduce yield.

However, these positive trends should be interpreted within the studied range, as excessive values may introduce limitations such as catalyst agglomeration, phase effects, equilibrium constraints, or side reactions. This is chemically reasonable, since the catalyst amount increases the availability of active sites, alcohol excess shifts the equilibrium toward ester formation, longer reaction time promotes conversion, and higher temperature enhances reaction kinetics within the studied range. Similarly, the negative acid-value contribution is consistent with neutralization and soap formation in base-catalyzed systems, although its effect may differ for acid-functional catalysts. In contrast, high acid value often reflects more challenging feedstock conditions that can hinder transesterification.

Surface area and mixing intensity showed slight net negative effects, while metal loading generally exhibited a negative contribution in the fitted model. These trends indicate that individual catalyst descriptors do not directly represent catalytic performance, as accessible active sites, pore accessibility, and metal dispersion may be more influential than bulk properties alone. This suggests that textural properties alone do not guarantee improved performance and should be considered together with catalyst chemistry and operating conditions. Feedstock FFA showed a modest positive association, whereas feedstock, metal type, and biochar source had more contextual, case-specific effects.

The different trends observed for FFA and acid value also highlight the influence of correlated descriptors and catalyst-specific effects, requiring cautious mechanistic interpretation. An important practical outcome of the explainability analysis is the identification of a compact set of variables that should be prioritized in future experiments and data reporting. As indicated in Fig. 14 and 15, catalyst amount, alcohol-to-oil ratio, reaction time, acid value, and temperature should be carefully measured and consistently reported for process optimization, as they exert the strongest influence on model predictions. Although surface area and metal loading showed comparatively lower importance, their nonzero contributions indicate that these catalyst descriptors should also be documented consistently.

The literature comparison in Table 9 shows strong agreement between the explainability results obtained in this study and prior biodiesel research. These comparisons serve as a chemical validation of the model interpretation; however, the main contribution lies beyond confirming well-known operating trends. Across review papers and recent data-driven studies, alcohol-to-oil ratio, catalyst amount, reaction time, and reaction temperature are repeatedly identified as the main variables controlling biodiesel conversion and yield.^36^ This is consistent with the present CatBoost and SHAP analyses, in which these variables formed the dominant group. The literature also strongly supports the negative role of acid value/free fatty acid content, since high acidity promotes soap formation, complicates separation, and lowers transesterification efficiency, especially in base-catalyzed systems.^37^

**Table 9:** Literature comparison supporting the major variables identified in this study

Study focus	Variables emphasized	Main findings	Agreement with our study	Ref.
Review of alcohol-to-oil molar ratio effects in biodiesel transesterification	Alcohol-to-oil ratio, alcohol type	Excess alcohol shifts equilibrium toward ester formation, increasing biodiesel yield	Supports the very high importance of the alcohol/oil molar ratio	36
Examining catalysts, feedstock characteristics, and process variables	Reaction time, catalyst concentration, alcohol/oil ratio, and reaction temperature	Temperature, catalyst loading, alcohol-to-oil ratio, and reaction time strongly influence biodiesel yield	Demonstrates the significance of temperature, catalyst quantity, alcohol/oil ratio, and reaction time	40
Review on improving heterogeneous catalysis for biodiesel production	Alcohol/oil ratio, catalyst, FFA, reaction time, temperature, agitation	Feedstock properties, catalyst, FFA content, and operating conditions jointly determine biodiesel production	Supports the ranking of major process variables and the smaller but measurable role of mixing intensity	41
Examining process parameters, catalysts, and feedstock characteristics	Feedstock properties, catalyst properties, and transesterification conditions	Biodiesel yield depends on feedstock characteristics, catalyst properties, and process conditions	Supports the combined interpretation of feedstock, catalyst, and operating-condition descriptors	39
Review of recent advances in transesterification for sustainable biodiesel production	FFA/acid value, pretreatment needed	High FFA requires pretreatment to prevent soap formation and yield loss	Strongly supports the negative SHAP effect of the acid value	37
Acid-value reduction study for high-FFA feedstock	Acid value/FFA	FFA > 1% hinders transesterification, promotes soap formation, and lowers biodiesel yield	Supports the strong negative influence of acid value in your model	42
Review of biochar-derived catalysts in (trans)esterification	Surface area, porosity, and surface acidic groups	Biochar catalyst performance depends on surface area, porosity, and active surface groups	Supports the non-zero contribution of surface area while explaining why it is not dominant alone	38
Review of catalysts used in biodiesel production	Metal loading/catalyst loading	Catalyst loading has an optimum range; excessive loading may reduce performance	Supports the finding that metal loading has a weaker, context-dependent effect	43
Data-driven analysis of biodiesel production factors	Feedstock type, catalyst quantity, alcohol/oil ratio, reaction time, temperature	Feedstock type and key operating parameters are consistently the most influential variables	Supports the observation that some categorical variables are contextual rather than globally dominant	44

In addition, previous studies on biochar- and carbon-based catalysts indicate that surface area, porosity, and active surface chemistry can influence biodiesel production, but textural properties alone do not always correlate directly with catalytic activity.^38^ This supports the present finding that surface area and metal loading contribute to prediction, but with lower global importance than the main operating variables. Overall, the comparison confirms that the feature-importance pattern identified by the present explainable ML framework is chemically reasonable and well aligned with established biodiesel literature.^39^ More importantly, the framework identifies descriptor limitations and potential interactions that conventional literature comparisons cannot resolve.

#### 4.4.2 Mechanistic insights revealed by explainable machine learning analysis

Although the importance of operating variables such as alcohol-to-oil ratio, catalyst amount, reaction time, temperature, and acidity agrees with established biodiesel knowledge, the contribution of explainable ML lies in revealing their conditional effects across heterogeneous catalyst–feedstock systems. Rather than indicating universal independent effects, the SHAP patterns suggest that biodiesel yield emerges from interactions among reactant availability, accessible catalytic sites, reaction kinetics, and feedstock characteristics.

For example, the high contribution of alcohol-to-oil ratio does not simply indicate that increasing alcohol always improves yield. Its effect depends on catalyst accessibility, phase behavior, and reaction conditions, where excessive alcohol may introduce dilution and separation penalties. Similarly, catalyst amount represents the availability of catalytic sites but does not directly measure catalytic activity, as the effective contribution depends on dispersion, accessibility, and interaction between catalyst particles and reactants.

The explainability analysis provides additional insight by identifying limitations of commonly reported catalyst descriptors. The lower contributions of surface area, metal loading, and catalyst identity do not imply weak chemical relevance; rather, they indicate that these descriptors incompletely represent catalytic functionality across studies. Properties such as accessible active-site density, acid/base strength, metal dispersion, pore accessibility, and surface chemistry are likely more transferable descriptors but are rarely reported consistently in literature datasets.

The contrasting effects of acid value and FFA further demonstrate the advantage of explainable ML over conventional feature ranking. While high acid value generally reduces yield in base-catalyzed systems through neutralization and soap formation, FFA contributions may vary depending on catalyst functionality and feedstock selection. Such relationships are difficult to identify using single-study optimization because they emerge from interactions among catalyst class, feedstock characteristics, and operating conditions.

Therefore, the main contribution of the explainability analysis is not the confirmation of well-known biodiesel parameters, but the identification of which relationships remain transferable across diverse catalytic systems and which descriptors require refinement. These insights provide a basis for developing more mechanistically informed catalyst databases and guiding future experimental designs.

#### 4.4.3 Study limitations

Although the proposed explainable machine learning framework demonstrates strong predictive performance and reliable generalization capability, a few limitations should be acknowledged to ensure transparency and scientific rigor.

First, the dataset was compiled from multiple literature sources, where experimental conditions, including catalyst synthesis methods, reactor configurations, feedstock characteristics, and analytical procedures, are not fully standardized. This heterogeneity improves dataset diversity and supports the learning of generalized relationships; however, it also introduces variability that is not entirely represented by the available input features. Therefore, residual errors and lower importance of some catalyst-related variables may partly reflect unobserved experimental heterogeneity rather than limited chemical relevance.

Second, although literature-based data augmentation through curve digitization significantly increased dataset size and improved model training, the augmented samples are derived from reported experimental trends rather than newly generated controlled experiments. However, this limitation is partially mitigated by the adopted validation strategy, which includes both an internal train/test split and independent external experimental validation conducted on newly measured data. Third, while the model incorporates key process and catalyst descriptors available across the literature, some detailed physicochemical properties of catalysts (*e.g.*, pore structure, surface chemistry, and active-site distribution) were not consistently reported and therefore could not be included in the feature space. The absence of descriptors such as active-site characteristics, metal dispersion, and surface functionality limits deeper catalyst–activity interpretation.

Importantly, these limitations do not affect the validity of the proposed framework. Rather, they reflect the inherent challenges of literature-based datasets and highlight the need for integrated experimental-data-driven approaches. The inclusion of independent experimental validation further strengthens the robustness and practical applicability of the developed model.

#### 4.4.4 Practical implications and applicability

The proposed explainable machine learning framework provides practical value for catalyst screening, biodiesel yield prediction, and process optimization, thereby reducing the need for extensive experimental trials. Its role is to prioritize experiments rather than replace experimental validation, enabling efficient screening of promising catalyst–process combinations. By identifying the most influential process and catalyst descriptors, the model can support experimental design and accelerate decision-making in catalyst development and process optimization. Furthermore, SHAP analysis provides insight into the factors driving individual predictions and can guide future experiments toward unresolved mechanisms and missing catalyst descriptors.

The applicability domain of the developed models is limited to metal-doped biochar and activated carbon-based catalyst systems, as well as to the range of operating conditions represented in the training dataset, including reaction temperature, time, alcohol-to-oil ratio, and catalyst loading. Predictions involving unseen catalyst chemistries, extreme conditions, or unfamiliar descriptor combinations should be treated cautiously, even when individual variables fall within the observed ranges. Predictions outside this domain should be interpreted with caution, as extrapolation beyond the available data distribution may reduce reliability. Therefore, the framework should be used as a decision-support tool with uncertainty awareness rather than a replacement for mechanistic understanding. Within these boundaries, the framework provides a reliable and interpretable tool for understanding biodiesel production behavior and supporting data-driven catalyst design and process optimization.

## 5. Conclusion and future work

This study developed an explainable machine learning framework for biodiesel production over metal-doped biochar and activated carbon catalysts, integrating both yield prediction and feature importance analysis using a literature-derived dataset. The work combined dataset preprocessing, curve digitization for literature-based data reconstruction, comparative model training, study-level holdout evaluation, and explainability analysis to achieve strong predictive performance and process-level interpretation. Unlike conventional predictive studies that focus primarily on accuracy, the proposed framework connects prediction with mechanistic interpretation by identifying the variables governing catalyst–process interactions. A key aspect of this study was substantial dataset expansion through curve digitization, which increased data resolution while preserving the original experimental studies. To prevent data leakage, splitting was performed at the study level, with the majority of studies used for training and validation and a small subset reserved as an unseen holdout set.

Six models were benchmarked: linear regression, SVM, RF, CatBoost, neural network, and PINNs. The results confirmed that nonlinear ML models are substantially more suitable than linear regression for biodiesel-yield prediction in this heterogeneous literature-based system. On the unseen dataset, the neural network achieved the best performance (*R*^2^ 0.95, RMSE 3.27%), followed closely by CatBoost (*R*^2^ 0.95, RMSE 3.45%). SVM and RF also performed well (*R*^2^ > 0.94). In contrast, the PINN benchmark showed lower accuracy (*R*^2^ 0.71, RMSE 8.48%), while linear regression performed worst overall (*R*^2^ 0.24, RMSE 13.80%), confirming the strongly nonlinear nature of biodiesel-yield prediction. Model robustness was further confirmed through external validation using a newly published independent experimental dataset. The neural network achieved an RMSE of 2.544% and an *R*^2^ of 0.955, while the CatBoost model yielded an RMSE of 2.721% and an *R*^2^ of 0.949. The consistent performance of different nonlinear models indicates that the retained descriptors capture transferable relationships within the represented catalyst and operating domain, although broader validation across diverse catalyst families remains necessary.

The explainability results showed that catalyst amount, alcohol-to-oil ratio, reaction time, reaction temperature, and acid value are the most influential variables controlling biodiesel yield. SHAP analysis further indicated that higher catalyst amount, alcohol-to-oil ratio, reaction time, and temperature generally increase the predicted yield, whereas higher acid value tends to reduce it. Importantly, the analysis demonstrates that these variables should not be considered independent factors, as their effects depend on catalyst characteristics, feedstock properties, and operating regimes. These findings are chemically meaningful and provide useful guidance for catalyst screening, process optimization, and experimental design. Overall, the study demonstrates that explainable ML can serve as an effective tool for biodiesel-yield prediction when experimental data are limited but literature information is abundant. Its main value lies in accelerating hypothesis generation and experimental prioritization rather than replacing mechanistic investigation.

Future work should focus on incorporating more consistently reported catalyst physicochemical properties, expanding the database with additional experimental studies, improving the physics-informed benchmark, and integrating the predictive framework with optimization tools for catalyst and process design. Future datasets should include mechanistically relevant descriptors such as active-site density, surface functionality, metal dispersion, pore accessibility, and catalyst stability to enable deeper structure–activity understanding. Furthermore, uncertainty quantification and multi-objective optimization considering yield, energy demand, catalyst recyclability, and environmental impact will be essential for developing practical sustainable biodiesel design strategies. This would further strengthen the practical utility of the proposed framework for sustainable biodiesel-production research.

## Author contributions

Menna Ebrahim: conceptualization, data curation, formal analysis, investigation, methodology, software, validation, visualization, writing – original draft, and writing – review and editing. Mostafa Mahmoud: data curation, investigation, methodology, software, validation, visualization, and writing – review and editing. Fatma H. Ashour: conceptualization, formal analysis, funding acquisition, investigation, project administration, resources, supervision, validation, and writing – review and editing. Osama A. Ibrahim: data curation, investigation, methodology, software, supervision, validation, visualization, and writing – review and editing. Nourhan H. Khashaba: conceptualization, data curation, investigation, methodology, resources, supervision, validation, visualization, and writing – review and editing.

## Conflicts of interest

The authors state that they have no financial conflicts of interest or personal relationships that could have influenced the research presented in this paper.

## Supplementary Material

RA-016-D6RA05362A-s001

## Data Availability

The data used and analyzed during the current study are available from the corresponding author upon reasonable request. Supplementary information (SI) is available. See DOI: https://doi.org/10.1039/d6ra05362a.
