# Supplementary material for: A catalyst-aware explainable machine learning framework for biodiesel yield prediction over metal-doped biochar and activated carbon catalysts
Source: RSC Adv. 2026 Aug 5;16(39):44973–97. doi: 10.1039/d6ra05362a (PMC13440591; doi:10.1039/d6ra05362a)
Supplement: Supplementary file 1 [file RA-016-D6RA05362A-s001.pdf]

Supplementary Information (SI) for RSC Advances.
This journal is © The Royal Society of Chemistry 2026

Supplementary Information

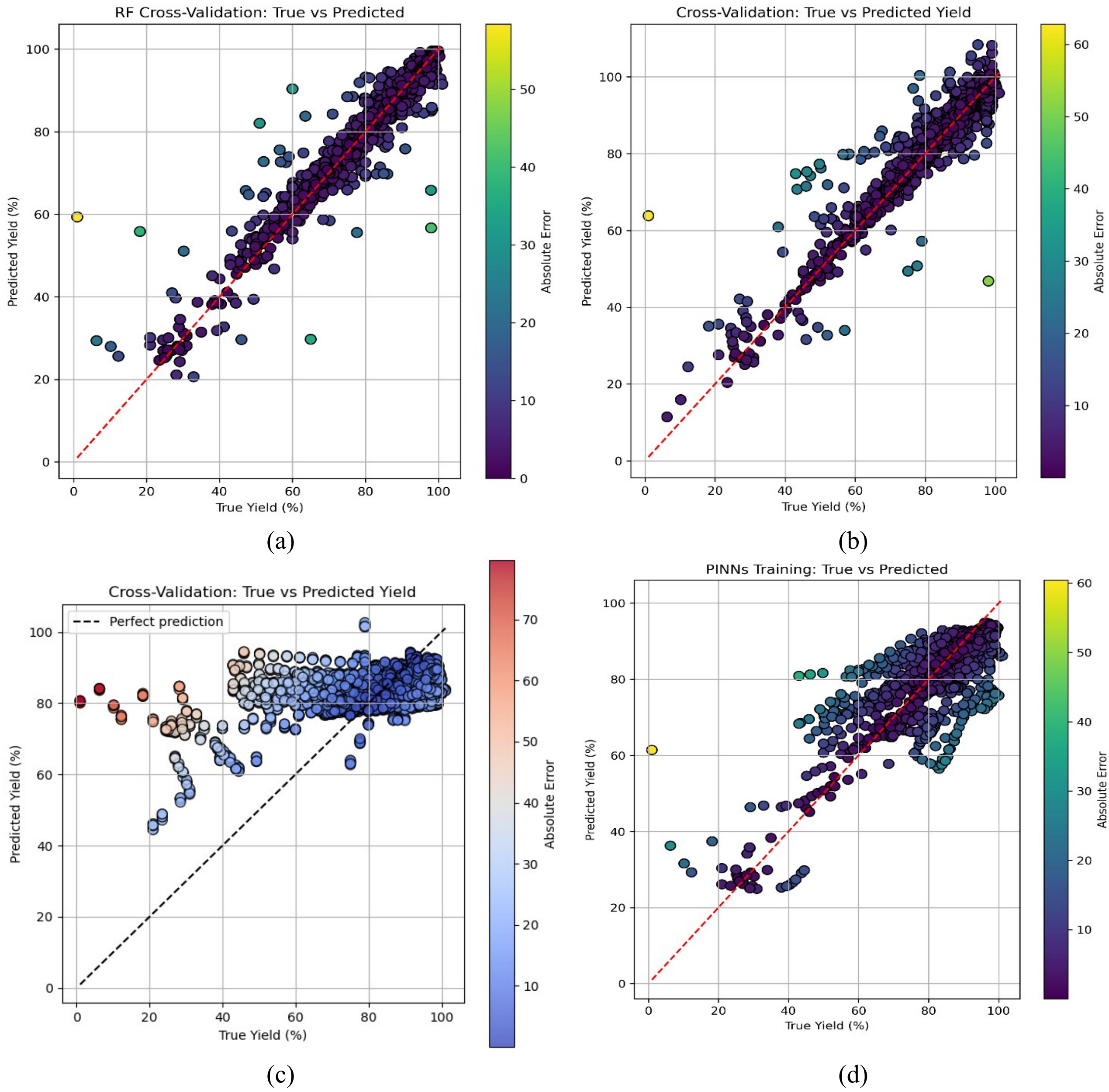

Fig. S1. True-versus-predicted training results for the developed models: (a) RF, (b) SVM, (c) Linear, and (d) PINNs.

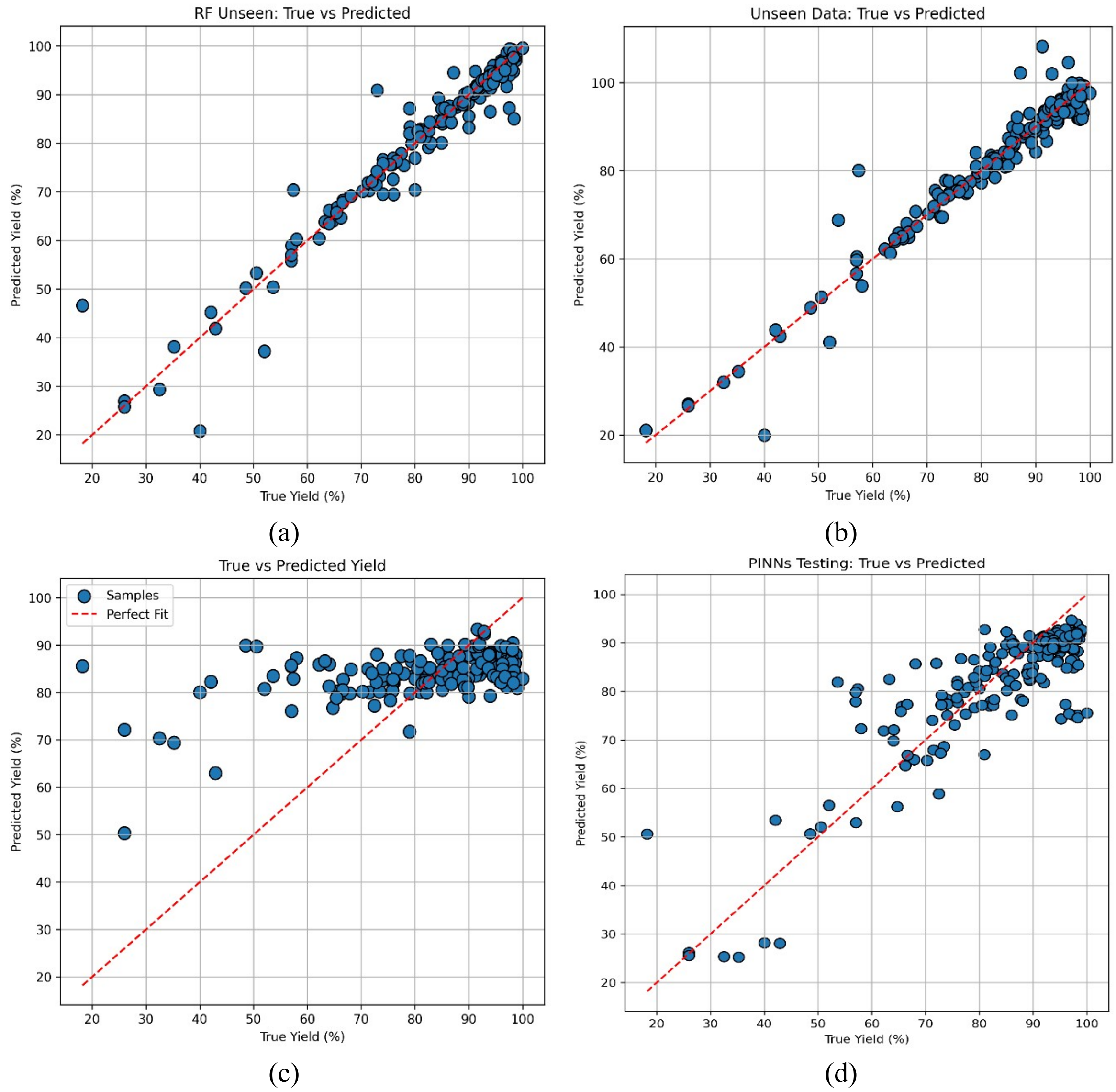

Fig. S2. Comparison of predicted versus actual yields on the unseen dataset for the developed models: (a) RF, (b) SVM, (c) Linear, and (d) PINNs.

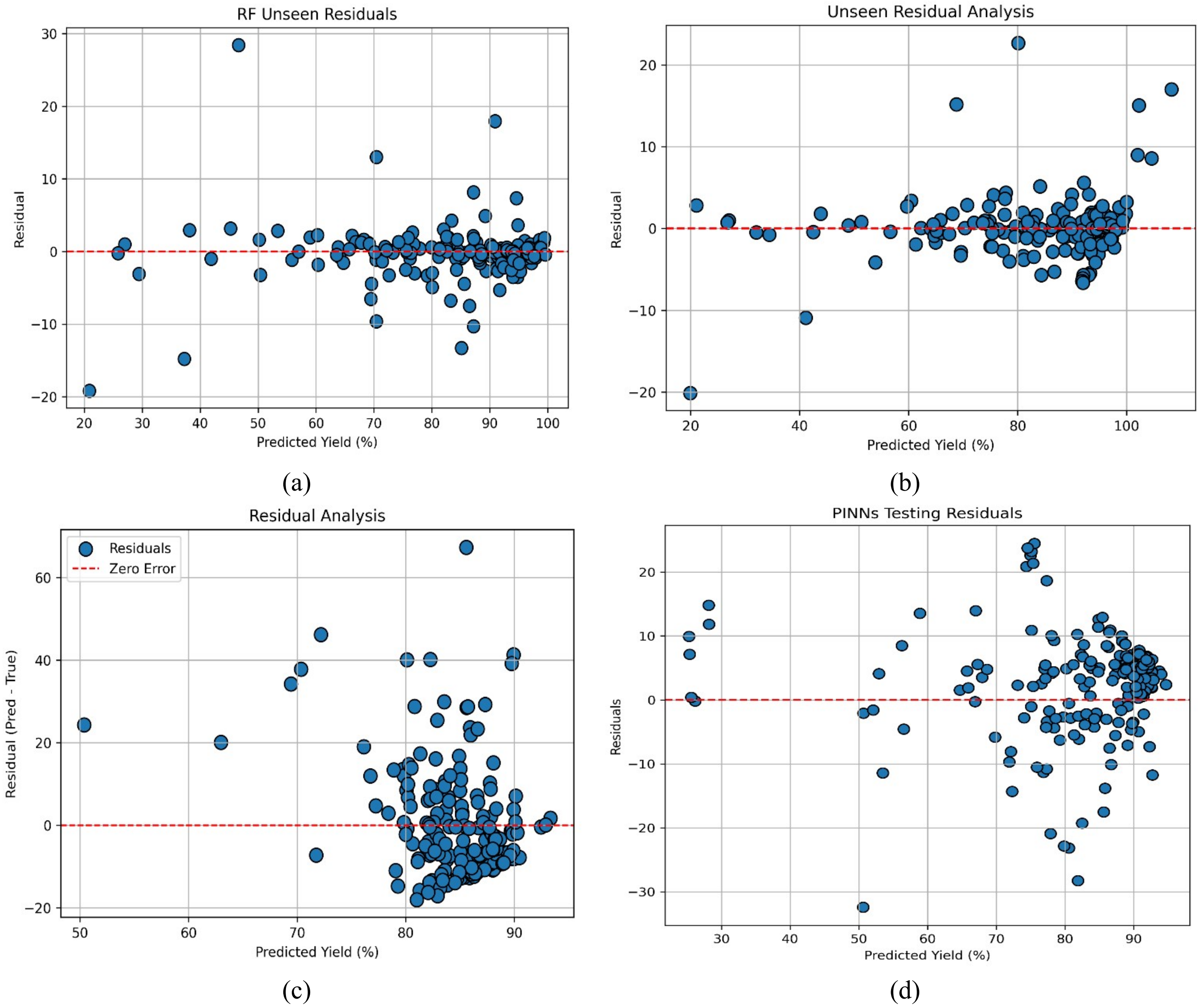

Fig. S3. Residual distribution of prediction errors on the unseen dataset for the developed models: (a) RF, (b) SVM, (c) Linear, and (d) PINNs.
